# Molecular targeting therapies for neuroblastoma: Progress and challenges

**DOI:** 10.1002/med.21750

**Published:** 2020-11-06

**Authors:** Atif Zafar, Wei Wang, Gang Liu, Xinjie Wang, Wa Xian, Frank McKeon, Jennifer Foster, Jia Zhou, Ruiwen Zhang

**Affiliations:** ^1^ Department of Pharmacological and Pharmaceutical Sciences, College of Pharmacy University of Houston Houston Texas USA; ^2^ Drug Discovery Institute University of Houston Houston Texas USA; ^3^ Department of Pharmacology and Toxicology, Chemical Biology Program University of Texas Medical Branch Galveston Texas USA; ^4^ Department of Biology and Biochemistry, Stem Cell Center University of Houston Houston Texas USA; ^5^ Department of Pediatrics, Texas Children's Hospital Section of Hematology‐Oncology Baylor College of Medicine Houston Texas USA

**Keywords:** clinical, neuroblastoma, preclinical, signaling pathway, targeted therapy

## Abstract

There is an urgent need to identify novel therapies for childhood cancers. Neuroblastoma is the most common pediatric solid tumor, and accounts for ~15% of childhood cancer‐related mortality. Neuroblastomas exhibit genetic, morphological and clinical heterogeneity, which limits the efficacy of existing treatment modalities. Gaining detailed knowledge of the molecular signatures and genetic variations involved in the pathogenesis of neuroblastoma is necessary to develop safer and more effective treatments for this devastating disease. Recent studies with advanced high‐throughput “omics” techniques have revealed numerous genetic/genomic alterations and dysfunctional pathways that drive the onset, growth, progression, and resistance of neuroblastoma to therapy. A variety of molecular signatures are being evaluated to better understand the disease, with many of them being used as targets to develop new treatments for neuroblastoma patients. In this review, we have summarized the contemporary understanding of the molecular pathways and genetic aberrations, such as those in MYCN, BIRC5, PHOX2B, and LIN28B, involved in the pathogenesis of neuroblastoma, and provide a comprehensive overview of the molecular targeted therapies under preclinical and clinical investigations, particularly those targeting ALK signaling, MDM2, PI3K/Akt/mTOR and RAS‐MAPK pathways, as well as epigenetic regulators. We also give insights on the use of combination therapies involving novel agents that target various pathways. Further, we discuss the future directions that would help identify novel targets and therapeutics and improve the currently available therapies, enhancing the treatment outcomes and survival of patients with neuroblastoma.

**Abbreviations**
4PB: 4‐phenylbutyate
ADCC: antibody‐dependent cell‐mediated cytotoxicity
AKT: protein kinase B
ALCL: anaplastic large‐cell lymphoma
ALK: anaplastic lymphoma kinase
ASCL1: Achaete‐scute family bhlh transcription factor 1
ASCT: autologous stem cell transplantation
AURKA: Aurora kinase A
Bcl‐2: B‐cell lymphoma 2
BCL‐xL: B‐cell lymphoma‐extra large
BDNF: brain‐derived neurotrophic factor
BET: bromodomain and extra‐terminal domain
BIRC5: baculoviral IAP repeat containing 5
BM: basement membrane
BMP4: bone morphogenetic protein 4
BMPs: bone morphogenetic proteins
BRAF: v‐raf murine sarcoma viral oncogene homolog B1
BRD: bromodomain
CAM: chorioallantoic membrane
CAR: chimeric antigen receptor
CDK: cyclin‐dependent kinases
CIC: Capicua transcriptional repressor
CIN: chromosome instability
COG: Children's Oncology Group
CPC: chromosomal passenger complex
CQ: chloroquine
DFMO: difluoromethylornithine
DNMT: DNA methyltransferase
ECM: extracellular matrix
EFS: event‐free survival
EGFR: epidermal growth factor receptor
ERBB2: Erb‐b2 receptor tyrosine kinases 2
FDA: Food and Drug Administration
FGFR1: fibroblast growth factor receptor 1
FUS: focused ultrasound
FZD2: Frizzled class receptor 2
GD2: Disialoganglioside
GSK3β: glycogen synthase kinase 3β
HAT: histone acetyltransferase
HBP1: HMG‐box transcription factor 1
HDACi: histone deacetylase inhibitor
HDACs: histone deacetylases
HDC: high‐dose chemotherapy
HIF: hypoxia‐inducible factor
HIFU: high‐intensity focused ultrasound
IC: induction chemotherapy
ID2: Iinhibitor of DNA binding 2
INSM2: insulinoma‐associated 2
JAK2: Janus kinase 2
LTLD: lyso‐thermosensitive liposomal doxorubicin
MAPK: microtubule associated protein kinase
Mash1: mammalian achaete‐scute homolog 1
Max: MYC‐associated factor X
MCL‐1: myeloid cell leukemia 1
MDM2: mouse double minute 2 homolog
MDR: multidrug resistance
MEK: mitogen‐activated protein
MIBG: metaiodobenzylguanidine
MR: magnetic resonance
MRI: magnetic resonance imaging
MTD: maximum tolerated dose
mTOR: mammalian target of rapamycin
NANT: New Advances in Neuroblastoma Therapy
NB: neuroblastoma
NCCs: neural crest cells
NEPENTHE: Next Generation Personalized Therapy
NF1: neurofibromatosis type I
NGF: neural growth factor
NHLH2: Nescient helix‐loop‐helix protein 2
NMC: NUT Midline Carcinoma
Notch 1: Notch receptor 1
NTRK: neurotrophic tropomyosin receptor kinase
ODC1: ornithine decarboxylase 1
OS: overall survival
PDGFR: platelet‐derived growth factor receptor alpha
PDX: patient‐derived xenograft
PHOX2: paired‐like homeobox 2b
PI3K: phosphatidylinositol‐3‐kinase
PIK3CA: phosphatidylinositol‐4,5‐bisphosphate 3‐kinase catalytic subunit alpha
PKMT: lysine methyltransferases
PPTP: pediatric preclinical testing program
PTEN: phosphatase and tensin homolog
PTPN11: tyrosine‐protein phosphatase non‐receptor type 11
RAS: rat sarcoma
RIPK1/3: receptor‐interacting serine/threonine‐protein kinase 1/3
RP2D: recommended phase 2 dose
RSPO2: roof plate‐specific spondin 2
RTK: receptor tyrosine kinase
SNP: single nucleotide polymorphism
Sox10: SRY‐related HMG‐box gene 10
TCF3: transcription factor 3
TLRs: Toll‐like receptors
TME: tumor microenvironment
TNF: tumor necrosis factor
TRAIL: tumor necrosis factor (TNF)‐related apoptosis‐inducing ligand
TrK: tropomyosin receptor kinase
TWIST1: twist‐related protein 1
UTR: untranslated region
VEGF: vascular endothelial growth factor
WNT1: WNT family member 1
ZA: zoledronic acid

## 1 INTRODUCTION

Neuroblastoma (NB) is a heterogeneous solid tumor that arises in the sympathetic nervous system. NB tumors most commonly develop in the abdomen and are most frequently localized in the adrenal gland.^1^, ^2^ NB tumors account for 7%–8% of childhood malignancies, and approximately 650 NB patients are diagnosed each year in the United States. However, NB accounts for approximately 15% of all pediatric cancer deaths. While the survival for patients with low‐ and intermediate‐risk disease approaches 100%, the 5‐year survival rate for high‐risk NB patients is less than 50%.^2^, ^3^, ^4^, ^5^, ^6^ There are some ethnic differences in NB, with the disease being more prevalent in those with European ancestry, and African‐American children tending to exhibit higher‐risk disease.^7^ NB tumors have also been classified as an embryonic tumor, because evidence suggests that such tumors originate from neural crest cells (NCCs) during fetal development.^8^

NB develops from the cells of the sympathetic nervous system, particularly sympathoadrenal progenitor cells, which differentiate into adrenal chromaffin cells and sympathetic ganglion cells.^5^ The transformation of sympathoadrenal precursors into sympathetic ganglia and adrenal chromaffin cells requires several factors, including overexpression of neural growth factor (NGF) and MYCN, SRY‐related HMG‐box gene 10 (Sox10) and mammalian achaete‐scute homolog 1 (MASH1) induced by bone morphogenetic proteins (BMPs).^9^ The transformation of persistent resting progenitor cells into NB cells requires anaplastic lymphoma kinase (ALK) mutations and MYCN amplification, and involves transcription factors including Sox11, nescient helix‐loop‐helix protein 2 (NHIH2), Twist‐related protein 1 (TWIST1), achaete‐scute family bhlh transcription factor 1 (ASCL1), insulinoma‐associated 2 (INSM2), and transcription factor 3 (TCF3).^9^ Several transcriptional regulators are involved in deciding the fate of cells with sympathetic lineages, such as MASH1, inhibitor of DNA binding 2 (ID2), dHAND, hypoxia‐inducible factor (HIF), and paired‐like homeobox 2b (PHOX2), all of which likely play roles in the pathogenesis of NB.^10^, ^11^, ^12^, ^13^, ^14^, ^15^, ^16^ Elevated levels of N‐Myc protein produced due to MYCN amplification play an important role in the pathogenesis of NB. The MYCN locus encodes MYCNOS (antisense transcript), and this encodes N‐CYM.^17^ Inhibition of GSK3β (glycogen synthase kinase 3β)‐driven N‐Myc degradation leads to N‐CYM, stabilizing N‐Myc.^5^ ALK also plays a significant role in the transformation of sympathoadrenal cells into NB cells. The expression of ALK correlates with an inferior prognosis.^18^, ^19^ Overall, it is thought that activated ALK collaborates with MYCN to markedly accelerate NB growth.^20^

NB has been divided into four major stages; localized stages L1 and L2, disseminated stage M, and disseminated stage MS, which occurs in patients younger than 18 months of age. Various prognostic parameters have been used to classify NB tumors, including the degree of differentiation, presence or absence of stroma, mitosis‐karyorrhexis index, patient age, NB stage, histological category, MYCN oncogene status, DNA ploidy, and chromosome 11q status.^21^, ^22^ Further, these parameters also help to classify patients into four groups based on their risk of death: (i) very low, (ii) low, (iii) intermediate, and (iv) high‐risk.^23^

Patients with low‐risk NB have a favorable prognosis and a 5‐year survival rate of more than 90%.^9^ However, 60% of patients have high‐risk NB, and the prognosis of treatment in such patients remains poor.^9^ Patients categorized as having low‐risk NB are typically provided minimal therapy, and some children are curatively treated by surgery alone, or may experience spontaneous tumor regression.^5^, ^24^, ^25^ Milder chemotherapy is administered to patients in the intermediate‐risk group, and they may also be treated by removing the remaining tumor mass.^26^ The current standard treatment for high‐risk NB includes three treatment blocks—(i) induction, (ii) consolidation, and (iii) maintenance.^27^ Induction chemotherapy (IC) aims to reduce the tumor by shrinking it and also reducing the risk of metastasis via chemotherapy and surgery.^28^, ^29^ The consolidation block involves the administration of HDC (high dose chemotherapy) accompanied by ASCT (autologous stem cell transplantation) and radiotherapy.^28^, ^29^ Maintenance involves immunotherapy using anti‐disialoganglioside (GD2) monoclonal antibody (mAb) with cytokines and differentiation therapy using 11‐cis retinol.^28^, ^29^ The IC generally includes platinum compounds (carboplatin, cisplatin), etoposide, cyclophosphamide, and vincristine (COJEC)^1^, ^5^, ^25^, ^30^, ^31^, ^32^, ^33^, ^34^ and in North America also includes topoisomerase inhibitors (topotecan) and anthracyclines.^35^, ^36^ However, the response to IC is not sufficient in 1/3 of children with high‐risk NB.^34^, ^37^ During induction therapy, surgery is performed to resect primary tumor tissue.^38^, ^39^ The consolidation phase can provide improved event‐free survival (EFS), especially in NB patients who have undergone tandem ASCT.^6^

Approximately half of high‐risk patients do not respond to the first‐line therapy protocol or relapse in the first 2 years after treatment.^34^, ^37^, ^40^, ^41^ The outcome for high‐risk NB patients is very poor, with a 5‐year survival rate of less than 50%.^25^ In addition, the response to current standard treatments is highly heterogeneous, varying from total regression to the development of multi‐drug resistance and severe toxicities.^1^, ^42^ NB is a complex disease that exhibits biological, clinical, morphological, and genetic heterogeneity, making it arduous to develop a successful universal therapy.^27^, ^43^, ^44^ The high tumor heterogeneity, drug resistance, and severe toxicities associated with standard treatment in children all lead to relatively poor outcomes for NB treatment. It is also important to note that oncology drugs have the lowest LOA (likelihood of approval) from phase I (6.7%) compared with drugs used for other diseases (allergy, dermatology, urology, autoimmune disease, and ophthalmology).^45^ Complicating matters, the current treatments approved for NB have limited targeted specificity.^26^ Thus, efforts should be strengthened to understand the tumor biology and develop new, more effective therapies for patients with NB.

Significant progress has been made to comprehend the molecular mechanisms involved in the etiology and pathogenesis of NB, and these investigations have identified new therapeutic targets. In particular, genome‐wide association studies, transcriptomics, genome sequencing, and high‐throughput genome analysis have revealed genetic alterations and disrupted pathways that are responsible for NB growth and development. Many of these are being tested as druggable targets for patients with NB. The use of molecular targeted therapy focused on genomic aberrations and disrupted pathways represents a new approach for the treatment of NB that may result in improved efficacy and reduced toxicity. This review provides an overview of the current state‐of‐the‐art molecular understanding of the development and progression of NB, with a particular emphasis on genetic aberrations and disrupted molecular pathways. As noted, several excellent reviews have been published, covering recent advances made in pathogenesis, diagnosis, and clinical management of NB and interested readers are referred to those excellent publications.^4^, ^9^, ^22^, ^29^, ^42^, ^46^, ^47^, ^48^, ^49^, ^50^, ^51^, ^52^, ^53^, ^54^, ^55^, ^56^, ^57^, ^58^, ^59^, ^60^, ^61^, ^62^, ^63^, ^64^, ^65^, ^66^, ^67^, ^68^, ^69^, ^70^, ^71^, ^72^ In this review, we will emphasize how these advances in knowledge about molecular pathogenesis can be translated to developing molecular targeted therapies for NB management, especially personalized therapies. We also attempt to provide insights into the promise of combination therapy using inhibitors that target many different pathways and their investigation in clinical trials. Further, we point out the future directions that should be taken to improve or develop effective targeted therapy to improve the survival rates of patients with NB while reducing treatment‐associated toxicity.

## 2 MAJOR MOLECULAR PATHWAYS INVOLVED IN NB TUMORIGENESIS

**Figure 1:**
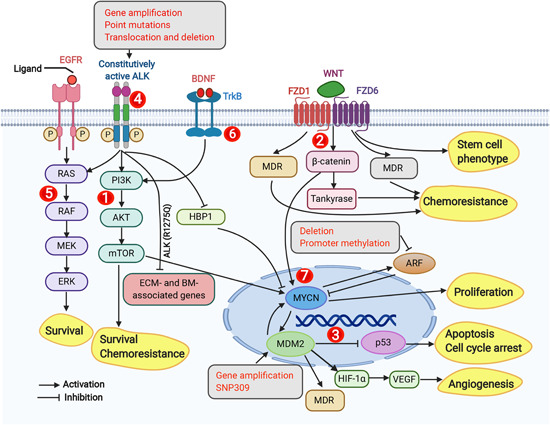
Overview of the molecular signaling pathways implicated in neuroblastoma. The signaling pathways described to play a role in neuroblastoma cells are (1) PI3K/AKT/mTOR pathway—promotes NB cell survival and chemoresistance; (2) Wnt signaling, which is involved in drug resistance, stemness, and increases MYCN levels; (3) p53‐MDM2 pathway, where MDM2 inhibits p53 activity, promotes angiogenesis, increases MYCN translation, and promotes drug resistance. Single nucleotide polymorphisms (i.e., SNP309) and gene amplification increase MDM2 expression. In the p53‐MDM2 pathway, activated p53 is involved in apoptosis and growth arrest; (4) ALK signaling activates PI3K/AKT/mTOR, RAS‐MAPK, and MYCN expression, and an ALK(R1275Q) mutant inhibits the expression of BM‐ and ECM‐associated genes; (5) RAS‐MAPK signaling promotes the survival of neuroblastoma cells and is activated by EGFR signaling; (6) TrkB signaling activates the PIK/AKT/mTOR signaling; (7) MYCN signaling promotes NB cell proliferation and activates MDM2 expression. Gray boxes in the figure represent genetic aberrations (i.e., gene amplification, point mutations, translocations, and deletions) and promoter methylation, and yellow boxes represent downstream biological phenotypes (i.e., survival, chemoresistance, angiogenesis, apoptosis, cell cycle arrest, proliferation, and stemness) in neuroblastoma cells. AKT, protein kinase B; ALK, anaplastic lymphoma kinase; BDNF, brain‐derived neurotrophic factor; BM, basement membrane; ECM, extracellular matrix; EGFR, epidermal growth factor receptor; ERK, extracellular signal‐regulated kinase; FZD1, frizzled‐1; FZD6, Frizzled‐6; HBP1, HMG‐Box transcription factor 1; HIF, hypoxia‐inducible factor; MDR, multidrug resistance; MDM2, mouse double minute 2 homolog; MEK, mitogen‐activated protein; mTOR, mammalian target of rapamycin; NB, neuroblastoma; PI3K, phosphatidylinositol‐3‐kinase; RAS, rat sarcoma; TrKB, tropomyosin receptor kinase B; VEGF, vascular endothelial growth factor [Color figure can be viewed at wileyonlinelibrary.com]

Recent research has been focused on identifying the molecular mechanisms involved in the NB pathogenesis. Ongoing investigations have identified several signaling pathways required for the growth and progression of NB (Figure 1), or that contribute to the resistance of the disease to conventional treatments. In this section, we provide a comprehensive summary of the role of signaling pathways involved in the pathogenesis of NB.

### 2.1 phosphatidylinositol‐3‐kinase (PI3K)/protein kinase B (AKT)/mammalian target of rapamycin (mTOR) pathway

PI3K/Akt/mTOR pathway is an important pro‐survival signaling pathway which is activated in most NBs.^73^ Immunohistochemistry using a human tissue microarray consisting of 116 primary NB specimens has been performed to analyze the phosphorylation of Akt. The study has revealed that Akt phosphorylation at serine 473 (S473) and/or threonine 308 (T308) is a common event in NB tissue.^74^ Moreover, Akt is highly phosphorylated, with S473 phosphorylation being present in 61.2% of primary NBs, with T308 being present in 62.9% of primary NBs.^74^ Further, that study has also revealed that 66 out of the 116 samples (56.9%) are positive for both antibodies (p‐Akt [S473] and p‐Akt [T308]), while 5 of the 116 (4.3%) exhibit S473 phosphorylation but not T308 phosphorylation, and 7 of 116 (6%) are found to have T308 phosphorylation but not S473 phosphorylation.^74^ A small percentage (32.8%; 38 out of 116 NB tumors) have low phosphorylation at both the S473 and T308 sites.^74^ In the same study, strong phosphorylation (55.2%) of the S6 ribosomal protein (a target of mTOR) has been observed in NB samples.^74^ The study has also confirmed that there is a correlation between Akt phosphorylation and MYCN amplification, which is significant for Akt phosphorylation at T308 or at both sites (S473 and T308), but not S473 phosphorylation alone.^74^ A study by Johnsen et al.^75^ has shown that Akt expression could be detected in all NB primary samples, but nonmalignant adrenal medullas lack this Akt expression. In addition, it has been found that catalytic p110α and the regulatory p85α isoforms of PI3K are more highly expressed in NB cell lines and primary NB samples compared with normal adrenal gland tissue.^76^

The PI3K/Akt pathway is upregulated in many malignancies through several mechanisms, including deletions or mutations of components of the signaling cascade (PIK3CA—phosphatidylinositol‐4,5‐bisphosphate 3‐kinase catalytic subunit alpha or PTEN—phosphatase and tensin homolog).^74^ However, deletions of the PTEN tumor suppressor gene (an antagonist of PI3K signaling) affect a small fraction of NB tissues. For instance, a screening study of 45 NB patients has revealed that homozygous deletions of PTEN are only found in 5% of primary tumors (2 of 41).^77^ Further, mutation analyses of the PIK3CA gene have also shown that mutations are only present in 2 out of 69 NB samples (27 NB‐derived cell lines and 42 primary tumors).^78^ These results suggest that the frequency of genetic aberrations (PTEN and PIK3CA) is relatively low in NBs. Nevertheless, activating mutations in ALK are observed in hereditary NB.^79^, ^80^, ^81^, ^82^ A study by Osajima‐Hakomori et al.^83^ has shown that suppression of ALK by RNA interference (RNAi) significantly reduces Akt phosphorylation and decreases the survival of NB cells.

NBs have been characterized to exhibit abnormal receptor tyrosine kinase (RTK) activity, which occurs mainly due to mutation or overexpression of growth factor receptors or their ligands. This has been involved in increased activation of the PI3K/Akt/mTOR pathway.^84^ Further, various growth factors have been found to affect the PI3K/Akt/mTOR pathways in NB. In particular, BDNF (brain‐derived neurotrophic factor) is a growth factor that transmits its signal via tyrosine kinase receptor tropomyosin‐related kinase B (TrkB) and promotes survival, and also confers chemoresistance by engaging the PI3/Akt/mTOR pathway.^85^, ^86^ An exogenous supply of BDNF or ectopic expression of TrkB causes increased Akt phosphorylation, which is associated with decreased sensitivity towards DNA damaging agents.^85^, ^86^ In NB, the PI3/Akt/mTOR pathway has been found to affect several pathways/proteins to enhance the NB phenotype. For example, the PI3/Akt/mTOR pathway contributes to MYCN stabilization, and MYCN, in turn, contributes to several processes associated with malignancy, such as proliferation, angiogenesis, and altering metabolic programming.^87^, ^88^ A study by Chesler et al.^89^ has shown that the PI3/Akt/mTOR pathway inhibition leads to a decrease in the levels of N‐Myc protein in NB.

### 2.2 WNT signaling pathway

WNT/β‐catenin signaling has been found to be responsible for NB progression and development. Enhanced WNT/β‐catenin signaling augments the MYCN levels in non‐MYCN‐amplified NB cells.^90^ A study by Zhang et al.^90^, ^91^ has shown that silencing WNT family member 1 (WNT1) expression by RNAi reduced the viability of SH‐SY5Y NB cells. Another study by Zins et al.^92^ has shown that knockdown of frizzled class receptor 2 (FZD2) inhibits the proliferation of the NB SK‐N‐AS and SK‐N‐DZ cell lines, and reduces the WNT3A‐facilitated SK‐N‐DZ cell migration and WNT5A‐facilitated SK‐N‐AS cell migration. Wnt/β‐catenin signaling also plays a key role in the chemoresistance of NB cells. For instance, higher expression of FZD1 and multidrug resistance (MDR) is present in doxorubicin‐resistant NB cells compared with non‐resistant NB cells.^93^ Knockdown of FZD1 in LAN1 NB cells also reduces the expression of MDR1 and restores sensitivity to doxorubicin.^93^ WNT3A/roof plate‐specific spondin 2 (RSPO2) signaling plays a critical role in regulating cyclin D1, BMP4, and the phosphorylation of RB protein, suggesting that WNT3a signaling has a role in the differentiation or progression of NB.^94^ Additionally, FZD6 (a Wnt receptor) positive NB cells are resistant to doxorubicin, form neurospheres, and express elevated levels of Twist1 and Notch receptor 1 (Notch 1) (mesenchymal markers),^95^ implying that FZD6 can be used as a marker of NB with stem cell properties. These observations indicate that Wnt signaling represents a promising target for therapeutic interventions in NB.

### 2.3 p53‐mouse double minute 2 homolog (MDM2) pathway

The MDM2 oncogene is an E3 ubiquitin ligase that inhibits p53 activity.^96^ Under unstressed conditions, MDM2 binds to the transactivation domain of p53 and targets p53 for ubiquitination and subsequent degradation by the proteasome.^97^, ^98^, ^99^ Studies have shown that amplification of MDM2 is found in a variety of malignancies, including NB.^100^ In addition, elevated MDM2 levels exist in NB without MDM2 gene amplification, and this is due to the existence of a single nucleotide polymorphism (SNP) in the MDM2 promoter.^101^ In general, MDM2 overexpression in human cancers has been found to be correlated to a poor prognosis, and associates with metastasis and advanced stages of the disease.^102^ The enhanced MDM2 activity leads to attenuation of p53 activity, and thus results in increased tumor formation. In addition, MDM2 has oncogenic functions independent of p53 that have been reported to be involved in NB growth and progression. In particular, MDM2 binds directly to the 3′‐untranslated region (3′‐UTR) of vascular endothelial growth factor (VEGF) and increases VEGF messenger RNA (mRNA) stabilization and translation, which promotes NB growth.^103^ In addition, MDM2 binds MYCN mRNA in the 3′‐UTR and thereby increases the stability and translation of MYCN mRNA in NB cells.^104^ Elevated MDM2 has also been found to cause multidrug resistance in NB cells.^105^ Thus, the oncogenic potential of MDM2 makes it a potential target for anticancer therapy in NB cells.

### 2.4 ALK signaling pathway

NB tumor tissues express full‐length ALK and exhibit single‐base missense mutations in the kinase domain of ALK, which promote ligand‐independent signaling.^106^, ^107^, ^108^ Single‐base missense mutations have been found in sporadic as well as familial NB^79^, ^82^, ^109^; and the mutations at F1174L, R1275Q, and F1245C comprise around 85% of all ALK mutations present in NB.^109^ The most common mutations are R1275Q and F1174L, both of which are present within the kinase domain of ALK, and these mutations lead to ligand‐independent autophosphorylation of ALK and increased kinase activity.^81^, ^110^, ^111^ The activation of ALK in NB leads to enhanced survival, migration, and cell proliferation.^110^ Further, ALK mutations have been found to hyperactivate rat sarcoma (RAS)‐microtubule associated protein kinase (MAPK) signaling in NB, thus promoting the development of cancer.^61^ Both the wildtype and mutant forms of ALK induce MYCN transcription in NB cells.^112^ In fact, a study conducted by Berry et al.^113^ has demonstrated that ALK mutations potentiate the oncogenic activity of MYCN in NB cells.

Apart from ALK mutations, around 2%–3% of cases involve gene amplification, leading to increased expression of ALK protein and constitutive kinase activity.^83^, ^114^, ^115^ It is also interesting to note that ALK is coamplified with MYCN, as the two genes are in proximity at 2p23 and 2p24, respectively.^80^, ^109^, ^116^ A study by Chang et al.^117^ has demonstrated that high MYCN expression is present in 24 (39.3%) and ALK protein in 25 (41%) of the 61 NB tumors analyzed. A mechanistic study has indicated that ALK plays a role in the positive regulation of MYCN activity through suppression of HMG‐box transcription factor 1 (HBP1) expression in NB cells.^118^ Further, large deletions and translocations lead to truncation of the extracellular region of ALK, providing another mechanism of ligand‐independent ALK signaling in NB.^119^, ^120^, ^121^ At relapse, NB tumors have been found to exhibit an increased frequency of ALK mutations.^122^, ^123^, ^124^ In fact, deep sequencing has revealed that F1174 and R1275 ALK mutations are present during diagnosis in 10% of cases, and these mutations are undetected by Sanger sequencing.^125^ ALK (R1275Q) is an activating mutation found in sporadic as well as familial NB patients.^126^ It downregulates the expression of extracellular matrix (ECM) and basement membrane (BM)‐associated genes in NB tumors.^126^ In addition, tumors with ALK (R1275Q)/MYCN have been found to exhibit reduced ECM/BM‐related protein expression compared with tumors with MYCN overexpression alone.^126^ Likely due to these changes in ECM/BM proteins, enhanced metastasis and invasion have been found in ALK (R1275Q)/MYCN mice.^126^ In NB cells, several miRNAs have been found to regulate ALK protein expression. Both miR‐424‐5p and miR‐503‐5p downregulate the ALK expression levels and decrease cell viability in ALK‐positive NB cells.^127^ A phosphoproteomics analysis has shown that ALK also promotes NB growth via the JNK signaling pathway.^128^

### 2.5 RAS/MAPK signaling pathway

The RAS/MAPK pathway is involved in the growth and survival of various pediatric malignancies, including NB. It has been estimated that 3%–5% of patients have mutations at the genetic level in the RAS‐MAPK pathway, while relapsed tumors (~80%) contain genetic mutations associated with this pathway.^53^, ^63^ In relapsed NB, the activating mutations of the RAS‐MAPK pathway detected include mutations in neurofibromatosis type I (NF1), v‐raf murine sarcoma viral oncogene homolog B1 (BRAF), tyrosine‐protein phosphatase nonreceptor type 11 (PTPN11), fibroblast growth factor receptor 1 (FGFR1), KRAS, NRAS, HRAS, and ALK.^123^, ^129^ Other nonmutational mechanisms of MAPK pathway activation include signaling via the tyrosine kinase receptors epidermal growth factor receptor (EGFR), ALK, and Erb‐b2 receptor tyrosine kinases 2 (ERBB2).^63^ Further, it has been found that mutations in the capicua transcriptional repressor (CIC) gene activate the RAS‐MAPK pathway, and such activation is responsible for increasing the tumorigenicity of NB cells.^130^ The effect of mutations of ALK, RAS‐MAPK, RAS, NF1, or BRAF, and their relationship to the sensitivity to MEK inhibitors have also been discussed in the past. In particular, it has been found that a nanomolar concentration of mitogen‐activated protein (MEK) inhibitor is sufficient to cause cell cycle arrest in NB cell lines with mutated RAS or BRAF genes.^130^ On the contrary, cell cycle inhibition in NF1‐ and ALK‐mutated NB cells is less effective.^130^

## 3 PRECLINICAL STUDIES OF TARGETED NB THERAPY

### 3.1 Targeting genetic and protein aberrations for NB therapy

**Figure 2:**
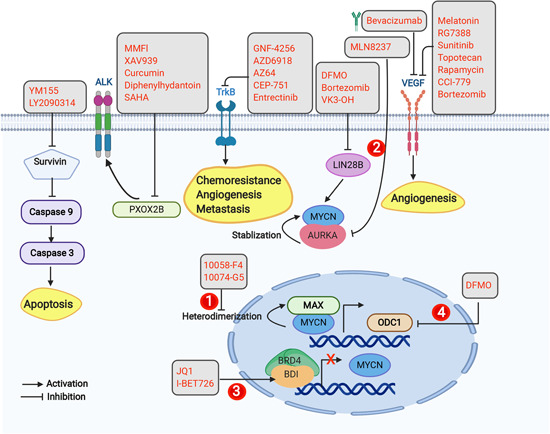
Targeted therapy involving genetic/protein aberrations in neuroblastoma cells. Some of the approaches employed under targeted therapy involve small molecule inhibitors of TrkB, VEGF, LIN28B, survivin, and Phox2b. Another approach is inhibition of MYCN using one of several strategies: (1) inhibition of MYCN/MAX heterodimerization; (2) inhibition of Aurora A kinase; (3) inhibition of bromodomain and extra‐terminal domain (BET) protein; and (4) inhibition of ODC1. Gray boxes represent inhibitors of survivin, PHOX2B, TrkB, LIN28B, VEGF, AURKA, MYCN/MAX heterodimerization, ODC1, and BET; yellow boxes represent downstream effects, including VEGF, TrkB, and caspase 3 activation. AURKA, Aurora A kinase; BDNF, brain‐derived growth factor; DFMO, difluoromethylornithine; Max, MYC‐associated factor X; MMF, mycophenolate mofetil; ODC, ornithine decarboxylase; Phox2b, paired‐like homeobox 2b; SAHA, suberoylanilide hydroxamic acid; TrKB, tropomyosin receptor kinase B; VEGF, vascular endothelial growth factor [Color figure can be viewed at wileyonlinelibrary.com]

As described above, ongoing investigations have identified numerous genetic and protein aberrations as potential therapeutic targets for NB (Figure 2), many of which have been evaluated in preclinical models of NB. Preclinical studies have identified several inhibitors (Figure 2) that can be pursued in clinical trials for NB patients.

#### 3.1.1 TrK inhibitors

TrK belongs to the neurotrophin receptor family and plays a critical role in NB biology. Elevated expression of TrkB is correlated with high‐risk NB and poor survival, while increased TrkA expression is correlated with lower‐risk NB and tumors that are prone to spontaneous regression.^131^, ^132^, ^133^, ^134^ In fact, patients with an advanced stage of NB and MYCN amplification have decreased TrkA expression.^132^, ^134^, ^135^ TrkB has been found to promote resistance to etoposide, doxorubicin, and cisplatin in NB.^86^ TrkB has also been reported to increase angiogenesis and metastasis.^136^, ^137^, ^138^ Thus, TrkB is a target for NB treatment. In preclinical models, Trk inhibitors GNF‐4256 and AZD6918 slow the growth of xenograft tumors, and combining a Trk inhibitor with chemotherapeutic drugs leads to significantly better effects compared with treatment with either agent alone.^139^, ^140^ AZ64, an inhibitor of neurotrophic tropomyosin receptor kinase (NTRK), has been found to inhibit TrkB, and enhance the efficacy of both local radiation and chemotherapy in a NB xenograft model.^141^ This data provides an indication that Trk inhibition may be a useful adjunct to existing chemotherapy. Further, CEP‐751 (KT‐6587) has been found to exhibit effective antitumor activity against NB cells and xenografts expressing elevated levels of TrkB.^142^, ^143^ Entrectinib (RXDX‐101) is another inhibitor of TrkA/B/C, and has been found to inhibit NB tumor growth, while entrectinib also augments the tumor growth inhibition of temozolomide when used in combination therapy in a xenograft mouse model.^144^

#### 3.1.2 MYCN inhibitors

The MYCN oncogene encodes a transcription factor that controls several cellular processes. MYCN gene amplification has been detected in 20%–30% of NB cases, and this amplification strongly correlates with the stage and aggressiveness of the disease.^145^, ^146^ A study conducted on 110 infants with stage 4s NB has indicated that patients with MYCN amplification have a worse survival compared with patients without MYCN amplification.^147^ Another study involving 2660 stage 1 or 2 NB patients has shown that patients diagnosed with tumors with MYCN amplification have a significantly worse EFS and inferior overall survival (OS) compared with patients without MYCN amplification.^146^ In a recent analysis of nearly 6000 patient samples, the presence of both homogenous and heterogenous MYCN amplification confers a worse EFS and OS compared with the prognosis of patients with wild‐type MYCN.^148^ These findings have supported that MYCN promotes angiogenesis, survival, and metastasis in NB, and inhibits immune surveillance.

Various strategies have been proposed to downregulate MYCN to decrease NB development, growth, and proliferation. One of the first strategies employed is targeting MYCN/MYC‐associated factor X (Max) interactions. After amplification, MYCN forms heterodimers with MAX to act as a transcription factor and promote NB growth.^149^, ^150^ Two compounds, 10058‐F4 and 10074‐G5, have been found to block heterodimerization, and treatment of MYCN‐amplified models of NB with these compounds induced differentiation and apoptosis in vitro conditions, and suppressed the growth of xenograft tumors.^151^, ^152^, ^153^

MYCN is stabilized by Aurora A kinase (AURKA) via protein‐protein interactions, which renders MYCN less prone to degradation by the proteasome.^53^ Thus, AURKA inhibition is a secondary approach to inhibit MYCN in NB cells. Treatment of IMR32 NB cells with MLN8237 (alisertib) (a specific aurora kinase inhibitor) induced cell senescence, cell growth inhibition, G2/M arrest, and MYC degradation, and induced inhibition of tumor growth in a xenograft mouse model.^154^ Another approach to inhibit MYCN includes the use of inhibitors that can inhibit bromodomain and extra‐terminal domain (BET) family of proteins.^53^ The BET proteins act as transcriptional regulators of many genes, including MYCN.^53^ BET inhibitors such as JQ1 cause bromodomain inhibition, which downregulates MYCN in NB cells, resulting in apoptosis and cell cycle.^155^ Further, GSK1324726A (I‐BET726) is involved in BET inhibition and decreases cell growth, induces cytotoxicity, and directly inhibits MYCN expression in NB cells.^156^

Yet another approach for targeting MYCN involves inhibition of ornithine decarboxylase 1 (ODC1).^53^ The ODC1 gene encodes an enzyme that catalyzes the rate‐limiting step of polyamine synthesis.^53^ Polyamines act as cationic chaperones to support the MCYN activity in NB cells via covalent and ionic mechanisms, and hence are responsible for maintaining the phenotype of NB.^53^ Difluoromethylornithine (DFMO), an ODC1 inhibitor, is an FDA‐approved treatment for trypanosomiasis. A study by Hogarty et al.^157^ has shown that disabling ODC1 by DFMO inhibited the proliferation of NB cell lines. The same study has also shown that the treatment of NB mouse model with DFMO is effective in delaying tumor initiation and enhancing the therapeutic effects of chemotherapy to increase the survival of mice with established tumors.^157^ Further, combined treatment using DFMO with celecoxib (a nonsteroidal anti‐inflammatory drug) have synergistic antitumor effects in NB models exhibiting ALK mutation, MYCN amplification, and *TP53* mutation with multidrug resistance.^158^

#### 3.1.3 Baculoviral IAP repeat containing 5 (BIRC5) and survivin inhibitors

The BIRC5 gene encodes human survivin, which is located on the long arm of chromosome 17 (q25).^159^ Advanced‐stage NB often exhibits a gain of the chromosomal 17q25 region,^160^ and the BIRC5 gene (present in this 17q25 region) is gained in 49% of NB tumors.^161^ Increased survivin expression is correlated with a poor prognosis in NB patients.^160^ In fact, the levels of survivin mRNA are higher in individuals older than 12 months, in advanced stages of disease (stages 3 and 4), and have a strong correlation with low levels of TrkA expression.^160^ The elevated levels of survivin expression in NB are also correlated with MYCN amplification.^160^ Survivin also increases glycolysis and resistance to treatment in NB.^162^, ^163^ In addition, survivin exerts antiapoptotic effects by inhibiting caspase 9 and enhancing resistance to apoptosis induced by staurosporine in NB cells.^164^ Survivin has also been found to provide resistance to immune‐ or drug‐mediated cell death.^165^ For example, a study of several NB cell lines has found that NB10, NB cell line that exhibits the least survivin expression, was the most sensitive to both TRAIL (tumor necrosis factor [TNF]‐related apoptosis‐inducing ligand) and etoposide induced cell death.^165^ On the contrary, the NB7 and NB16 cell lines, which have an abundance of survivin, were more resistant to TRAIL‐ and etoposide‐induced cell death.^165^ Survivin has also been found to cause the stabilization of the microtubules in the chromosomal passenger complex (CPC).^166^

Various inhibitors have been found to target survivin in preclinical studies of NB. For example, YM155 decreases the survivin expression, inhibits the proliferation of and induces apoptosis in NB SH‐SY5Y cells.^167^ The same study has also shown that reduced expression of survivin after treatment with YM155 is effective to sensitize SH‐SY5Y cells to cisplatin (chemotherapeutic agent), and induces tumor regression and apoptosis in SH‐SY5Y xenograft model.^167^ Research conducted by Kunnimalaiyaan et al.^168^ has demonstrated that LY2090314 (a GSK‐3 inhibitor) is capable for causing growth inhibition and inducing apoptosis in NB cells, and also reducing the survivin level. Withanolides (WA, WGA, WGB‐DA, WGA‐TA) have also been found to be cytotoxic to NB cells, potentially because they downregulate survivin in NB cells.^169^ Noscapine, a nontoxic natural compound, induces apoptosis via downregulation of survivin in both p53 wild type and null NB cells.^170^ Interestingly, the antidiabetic drug troglitazone also holds the capacity to sensitize NB cells to TRAIL‐induced apoptosis via downregulation of survivin.^171^

#### 3.1.4 VEGF inhibitors

VEGF is a 45 kDa dimeric glycoprotein that plays an important role in the formation of blood vessels (angiogenesis).^172^ Apart from the functions of VEGF in angiogenesis and vascular permeability, the autocrine signaling of VEGF plays a role in cancer stem cells, and the resistance of tumor cells to treatments.^173^, ^174^ The human VEGF‐A gene is positioned on chromosome 6 and contains eight exons.^175^ Alternate splicing of the VEGF gene generates several isoforms, including VEGF121, VEGF189, and VEGF165, which are expressed in different human tumors.^176^, ^177^ Among the different isoforms of VEGF, VEGF165 mRNA is the predominant isoform expressed in human NB cells.^178^ Increased expression of VEGF is found more frequently in advanced‐stage (stages 3 and 4) NB tumors compared with low‐stage (stages 1, 2, and 4S) tumors.^179^ Increased VEGF‐A levels have been observed in the serum and plasma of NB patients.^180^

The activity of several VEGF inhibitors has been investigated in preclinical models. For instance, melatonin has been found to inhibit angiogenesis in human SH‐SY5Y NB cells by downregulating VEGF.^181^ RG7388, an MDM2 inhibitor, causes tumor growth inhibition in p53 wildtype NB cells, and inhibits HIF‐1α/VEGF signaling, and alters angiogenesis.^182^ Sunitinib, a receptor tyrosine kinase inhibitor, has been found to impair NB growth and enhance the cytotoxic activity of chemotherapeutic drugs, and decreased MYCN and VEGF expression in NB cells.^183^ Topotecan (topoisomerase inhibitor), is capable of inhibiting HIF‐2α and HIF‐1α accumulation and also transcriptional activity, and thus inhibits VEGF expression in NB cells.^184^ mTOR inhibitors, namely rapamycin and CCI‐779, have been found to reduce VEGF‐A secretion, inhibit mTOR,and induce apoptosis and cell cycle arrest in NB cells.^75^ Bortezomib, a proteasome inhibitor, decreases cellular proliferation and induces cell cycle arrest, and also bortezomib treatment leading to a reduction of 76.3% of VEGF levels in treated tumors as compared with controls.^185^ Imatinib mesylate has also been found to inhibit the cellular growth of NB both in vitro and in vivo, and the inhibition of cellular growth is correlated with the decrease in expression of platelet‐derived growth factor receptor alpha (PDGFR), c‐kit, and VEGFR.^186^

#### 3.1.5 PHOX2B inhibitors

PHOX2B is the first genetic predisposition identified in NB. An estimated 6.4% of patients with hereditary NB have germline mutations of PHOX2B.^16^, ^187^, ^188^, ^189^ However, mutations in PHOX2B are rarely seen in the germline and tumor cells of sporadic NBs.^189^, ^190^ Loss‐of‐function mutations in the PHOX2B gene impede NB differentiation by disrupting calcium regulation.^191^ A study by Bachetti et al.^192^ has demonstrated that increased expression of PHOX2B in NB cells leads to an increase in ALK protein. In particular, PHOX2B contributes to the pathogenesis of NB by driving ALK gene expression by directly binding the ALK gene promoter.^192^ Elevated levels of Phox2b protein have been found in MYCN‐amplified NB cell lines, while no detectable Phox2b expression is found in NB cell lines with low MYCN expression.^193^ A real‐time qPCR study has shown higher PHOX2B expression in NB cell lines compared with normal tissues.^192^ A study by van Limpt et al.^16^ have described several different frameshift mutations of PHOX2B, such as 284‐291del8nt, 633‐670del38nt, 702‐714dup13nt, 721‐755del35nt, 721‐737dup17nt, and 721‐740del20nt in sporadic NBs. A study by Raabe et al.^189^ has described point mutations such as c.667G>C in a human NB‐derived cell line and c.299G>T in sporadic NB with multifocal primaries, as well as frameshift mutations (c.676delG and c.691_698dup8) in hereditary NB. Mutation of PHOX2B is correlated to RAS‐MAPK pathway activation in NB cell lines.^130^ It has also been found that one of the downstream targets of PHOX2B is the MSX1 homeobox transcriptional factor, and this transcriptional factor activates the Delta‐Notch pathway in NB.^194^

At the preclinical level, various molecules have been found to inhibit Phox2b in NB cells. For instance, mycophenolate mofetil decreases PHOX2B mRNA and protein expression in IMR32 NB cells, and induces Caspase 3/7 cleavage and apoptosis in NB cells.^195^ In another study by Di Zanni et al.,^196^ has demonstrated that curcumin, diphenylhydantoin, and suberoylanilide hydroxamic acid aree found to inhibit Phox2b in NB cells. XAV939 treatment has also been found to downregulate PHOX2A and PHOX2B in NB SH‐SY5Y cells, and the same study has also demonstrated that combination treatment with XAV939 significantly increases the sensitivity of IMR‐32 and SH‐SY5Y cells to doxorubicin treatment in two‐dimensional (2D), as well as three‐dimensional (3D), cultures.^197^

As mentioned above, PHOX2B activates the Delta‐Notch pathway in NB cells,^194^ and various studies have shown that Notch pathway activation induces NB cell growth and proliferation, suggesting that Notch is involved in the pathogenesis of NB.^198^, ^199^ A study by Funahashi et al.^200^ has shown that treatment with a Notch signaling antagonist inhibits angiogenesis and impairs tumor viability in a mouse model of NB, suggesting that Notch blockade or inhibition represents a potential therapy for NB. Several γ‐secretase inhibitors (GSIs) have been found to block Notch receptor cleavage, and thus GSIs have been developed and tested in Alzheimer's disease as Notch pathway inhibitors. For instance, a dipeptide analog called DAPT (a noncompetitive inhibitor of γ‐secretase) has been found to inhibit Aβ generation in the brain and plasma in an APP transgenic mouse model of Alzheimer's disease.^201^, ^202^ Likewise, indomethacin (a nonsteroidal anti‐inflammatory agent) is capable of lowering Aβ42 in in vitro and in vivo model systems by targeting the γ‐secretase complex.^203^ Based on these observations, GSIs are now being repurposed to test their efficacy against various human malignancies, including NB. For example, DAPT has been found to inhibit cellular growth, promote neuronal differentiation, and induce apoptosis in NB cells.^198^ Indomethacin has also been found to inhibit the growth of NB cells, and to enhance the chemosensitivity of NB.^204^

#### 3.1.6 LIN28B inhibitors

A SNP has been found in Lin28B and is strongly involved in the development of high‐risk NB.^205^ Research has shown that NB cells exhibit overexpression and amplification of LIN28B.^205^, ^206^ In NB, Lin28B increases the expression of N‐Myc via let‐7 miRNAs repression.^206^ Lin28 also promotes the stabilization of downstream AURKA and increases the oncogenic activity of RAN GTPase, hence promoting tumorigenesis.^207^ These features suggest that targeting LIN28 may be beneficial in treating NB. A study by Lozier et al.^208^ has shown that DFMO treatment reduces LIN28B protein levels in SMS‐KCNR, BE(2)‐C, and CHLA90 NB cells. The same study has also shown that the sensitivity to treatment with DFMO correlates with overexpression of LIN28B (BE(2)‐C>SMS‐KCNR>CHLA90).^208^ Bortezomib has also been found to inhibit LIN28B expression, and the combination of bortezomib and DFMO leads to more significant inhibition of LIN28B expression in NB cells than treatment with either agent alone.^209^ A derivative of vitamin K3 (VK3‐OH) has also been found to suppress LIN28B at the protein as well as mRNA levels in MYCN‐driven NB cells.^210^ Further, JQ1 and panobinostat have been found to synergistically downregulate the gene expression of LIN28B and protein expression of N‐Myc in NB cells.^211^ Thus, several different compounds exist that show potent effects against LIN28B.

### 3.2 Targeting signaling pathways for NB therapy

**Figure 3:**
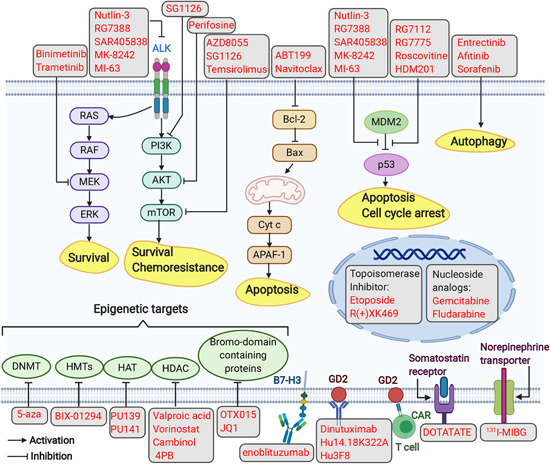
Targeted therapy in neuroblastoma. Several approaches to targeted therapy involve the following modalities: (1) small molecule inhibitors targeting signaling pathways (i.e., PI3K/AKT/mTOR, RAS‐MAPK, p53‐MDM2, Bcl‐2, and ALK); (2) chemical inhibitors inducing autophagy; (3) immunotherapy employing monoclonal antibodies targeting GD2 and B7‐H3, and using CAR T cells targeting GD2; (4) targeting epigenetic regulators; (5) radiopharmaceuticals targeting NET (^131^I‐MIBG) and the somatostatin receptor (DOTATATE); (6) targeted therapy based on topoisomerase inhibitors or nucleoside analogs. Gray boxes represent inhibitors of MEK, ALK, PI3K, AKT, mTOR, Bcl‐2, p53‐MDM2, epigenetic targets, and topoisomerases; compounds that act as autophagy inducers; nucleoside analogs; monoclonal Abs that target B7‐H3 or GD2; and radiopharmaceuticals targeting the somatostatin receptor and NET; yellow boxes represent downstream biological phenotypes (i.e., survival, chemoresistance, apoptosis, cell cycle arrest, and autophagy). AKT, protein kinase B; ALK, anaplastic lymphoma kinase; APAF‐1, apoptotic peptidase activating factor 1; Bax, BCL2‐associated X; Cyt c, cytochrome c; DNMT, DNA methyltransferases; ERK, extracellular signal‐regulated kinase; HAT, histone acetyltransferases; HDAC, histone deacetylases; HMT, histone methyltransferase; MDM2, mouse double minute 2 homolog; mTOR, mammalian target of rapamycin; PI3K, phosphatidylinositol‐3‐kinase; RAS, rat sarcoma [Color figure can be viewed at wileyonlinelibrary.com]

Extensive preclinical studies have been conducted to investigate the potential of targeting signaling pathways for the treatment of NB. Several potential inhibitors have been identified (Figure 3) employing in vitro and in vivo NB models.

#### 3.2.1 Targeting the p53‐MDM2 pathway in NB

In contrast to many other malignancies, NBs generally have intact and wild type p53.^212^, ^213^ Thus, reactivating the functional activity of wild type p53 by targeting the p53‐MDM2 pathway via MDM2 inhibitors may represent a favorable approach for the NB treatment. Various MDM2 inhibitors have been explored using preclinical NB models. For instance, nutlin‐3 (a p53/MDM2 antagonist) has been found to target the p53/MDM2 interaction and activate the p53 pathway in both chemosensitive and chemoresistant NB cells with wild‐type p53.^214^ The same study has also found that nutlin‐3 is effective against chemoresistant p53 wild type NB xenograft tumors in mice.^214^ Nutlin‐3a has also been found to increase the antitumor effects of chemotherapeutic drugs via rapid p53 stabilization in NB cells.^215^ A second generation nutlin, RG7388, has been developed, which exhibits increased efficacy and lower toxicity than nutlin‐3a. Treatment of NB cells with wild‐type p53 using RG7388 induces p53 activation and apoptosis.^182^ Other MDM2 antagonists which have been demonstrated anticancer activity in NB models include SAR405838 (MI‐77301),^216^ MK‐8242,^217^ MI‐63,^218^ RG7112,^219^ and RG7775.^220^ In addition, a study by Giustiniano et al.^221^ has shown that “compound 12” acts as a dual inhibitor of MDM2/p53 and MDM4/p53 complexes and enhances the p53 protein levels in human SHSY‐5Y NB cells. Roscovitine (seliciclib or CYC202) has also been found to inhibit cell viability, activate p53 and p53‐dependent genes (Bax, p21), and inhibit MDM2, N‐Myc, and Akt1 expression.^222^

#### 3.2.2 Targeting ALK signaling in NB

Aggressive efforts are underway to develop ALK inhibitors as targeted therapy for NB. Point mutations and amplification of ALK are oncogenic in nature in in vitro as well as in vivo conditions, leading to the constitutive phosphorylation of ALK and other downstream signaling molecules, which is important for the survival and proliferation of NB.^79^, ^80^, ^81^, ^82^ Interestingly, in contrast to aberrations like an amplification of MYCN, aberrant ALK is susceptible to inhibition by small molecules.^79^ At the preclinical level, it has been found that the R1275Q mutation and ALK amplification in NB cell lines are both sensitive to crizotinib, and regression of xenograft tumors has been observed following treatment.^223^ Another compound, CH5424802, inhibits the NB cellular growth, which expresses amplified ALK.^224^ Lorlatinib (PF‐06463922), a third‐generation ROS1 and ALK inhibitor, has been found to be effective against NB cells and against ALK‐mutated xenograft mouse models (both F1174L and F124C), as well as crizotinib‐resistant xenografts.^225^ In fact, lorlatinib has been found to demonstrate activity surpassing that of crizotinib.^225^ Moreover, lorlatinib has been demonstrated strong antitumor in xenograft models containing F1174L, F1245C, and R1275Q mutations.^225^

#### 3.2.3 Targeting the PI3K/AKT/mTOR and RAS‐MAPK pathways in NB

AZD8055 is a dual inhibitor of mTORC1‐mTORC2, and has been evaluated in preclinical NB models. It has been found to suppress growth and induce apoptosis in NB cell lines, and also decreases tumor growth in a xenograft model.^226^ With regard to targeting the RAS‐MAPK pathway, research has revealed that binimetinib, a MEK1/2 inhibitor, inhibits tumor growth and improves the survival of mouse models of NB.^123^ It has also been found that binimetinib exhibits synergistic effects with a ribociclib (cyclin‐dependent kinase [CDK]4/6 inhibitor), and suppresses the growth of tumors in a xenograft mice model.^227^

#### 3.2.4 Targeting antiapoptotic proteins and autophagy in NB

Among the numerous antiapoptotic proteins, research has been focused on the Bcl‐2 (B‐cell lymphoma 2) protein in NB cells. Testing new Bcl‐2 inhibitors at the preclinical level is a challenge, as most NB cell lines have low levels of Bcl‐2.^228^, ^229^ However, two Bcl‐2 inhibitors, venetoclax (ABT199) and navitoclax, have been examined in in vitro and in vivo models of NB.^53^ While venetoclax alone had moderate activity, when it was used in combination with alisertib (Aurora A kinase inhibitor), the activity was increased, with NB tumors exhibiting complete regression.^230^ Of note, it has been found that Bcl‐2‐dependent NB cells are sensitive to ABT‐199, while myeloid cell leukemia 1 (MCL‐1)‐dependent cells are completely resistant.^231^ B‐cell lymphoma‐extra large (BCL‐xL), a transmembrane protein present in the mitochondria, functions as antiapoptotic protein by preventing the release of mitochondrial contents, inducing caspase activation and ultimately apoptosis.^232^ Research has been carried out to investigate the combination of a BCL‐xL inhibitor with an Aurora kinase inhibitor in pediatric malignancies. Levesley et al.^232^ have demonstrated that when ABT‐263 (a BCL‐xL inhibitor) is used in combination with MLN8237 (an Aurora kinase inhibitor), it increases the sensitivity of MLN8237 cells, apparently via a BCL‐xL inhibition‐based mechanism. ABT‐263 promotes caspase‐dependent apoptosis and impedes cell division in MLN8237 human glioma and pediatric cell lines.^232^ The same study has also highlighted that BCL‐xL inhibition causes an increase in apoptosis when used in combination with chemotherapeutic agents.^232^ A study by Bate‐Eya et al.^233^ has demonstrated that combination treatment with an MCL‐1 inhibitor (1210477) and ABT199 leads to significant synergistic effects against NB cells. The above findings indicate that the use of combinations of targeted therapy may be necessary to overcome the resistance, including amyloid cell leukemia sequence MCL‐1‐dependent resistance, to Bcl‐2 and other targeted inhibitors.

Autophagy is associated with the development of resistance to molecular targeted therapies. The induction of autophagy is an important factor that promotes cancer cells to survive the stress induced by anticancer agents. For instance, entrectinib (an ALK inhibitor) treatment has been shown to induce autophagy in NB cells.^234^ The presence of the ALKF1174L mutation in SH‐SY5Y NB cells has been found to make them less sensitive to entrectinib compared with cells without this mutation.^234^ Treatment of a cell line harboring mutated ALKF1174L with a combination of entrectinib and chloroquine (CQ) (an autophagy inhibitor) has been found to increase cell death compared with entrectinib treatment alone.^234^ Afitinib and Sorafenib (receptor tyrosine kinase inhibitors), also induce autophagy in NB cells.^53^ As with entrecitinib, combination treatment using tyrosine kinase inhibitors with an autophagy‐blocking agent (either CQ or Spautin‐1) leads to significant increase in cell death in NB cells.^234^, ^235^

### 3.3 Targeting epigenetics in NB

In addition to genetic mutations and SNPs, epigenetic factors may also contribute to disease. Several different agents have been evaluated to target the epigenetic landscape in cancer cells. These include DNA methyltransferases (DNMTs), enzymes responsible for histone modifications (acetylation, methylation, and deacetylation) and chromatin readers.

#### 3.3.1 DNMTs

The expression of DNMTs is altered in NB.^236^ Among the different DNMTs, DNMT3A/B expression is higher in high‐risk NBs, and these enzymes are overexpressed in cisplatin‐resistant NB cells.^237^ A DNMT inhibitor, 5‐aza‐deoxycytidine (5‐aza or decitabine), has been tested in NB cells and found to reduce their proliferation and colony formation.^238^, ^239^ It has also been found that 5‐aza can potentiate the efficacy of currently available chemotherapeutic drugs (cisplatin, doxorubicin, and etoposide) in NB cells.^240^

#### 3.3.2 Histone modifications

Lysine methyltransferases (PKMT), a specific type of histone methyltransferase (which induce histone modifications), have been targeted in NB.^236^ In particular, BIX‐01294, an inhibitor of PKMT, decreases invasion and proliferation and induces apoptosis in NB cells.^241^ The addition of acetyl groups to histone lysine residues is another histone modification type, and is catalyzed by histone acetyltransferases (HATs).^242^ Various HAT inhibitors, such as PU139 (a HAT pan‐inhibitor) and PU141 (CBP and p300 selective inhibitors), have been investigated and found to reduce the growth of NB cells under both in vitro and in vivo conditions.^243^ Histone deacetylases (HDACs) appear to be related to the prognosis of NB. For example, HDAC8 and HDAC10 are overexpressed in NB, and their inhibition significantly reduces the proliferation of NB in vitro^244^, ^245^ and in vivo.^246^ Further, treatment with valproic acid (an HDACi) inhibits cellular proliferation and induces apoptosis and differentiation in NB cells.^247^, ^248^ Vorinostat (suberoylanilide hydroxamic acid or SAHA), is another HDACi, which results in G2/M phase arrest, followed by activation of the intrinsic apoptosis pathway.^249^ In addition, synergistic anticancer effects were observed when SAHA was combined with the proteasome inhibitor MG132 in NB SH‐SY5Y cells.^250^

As discussed previously, MYCN amplification is one of the important genetic aberrations associated with NB. HDACs have been found to take part in crosstalk with MYCN in NB. For instance, MYCN directly induces the transcription of SIRT1 (class III HDACs), and pharmacological inhibition via cambinol (a SIRT1 inhibitor) effectively reduces the growth of tumors in an MYCN‐driven transgenic mouse model of NB.^251^

#### 3.3.3 Chromatin readers

Chromatin readers identify histone modifications and recruit other proteins to the modification site to initiate or inhibit transcription. These readers include bromodomain‐, tudordomain‐, and chromodomain‐containing proteins. Among these readers, bromodomain (BRD)‐containing proteins can perform several functions, such as chromatin remodeling, histone activation, and transcriptional activation.^252^ The effects of BRD inhibition in NB have been evaluated using the BET inhibitor JQ1. The treatment effectively reduces the MYCN levels and cellular growth, and induces apoptosis in vitro as well as in vivo NB models.^155^, ^253^ In addition, when JQ1 is used in combination with panobinostat (HDACi), there is synergistic growth inhibition and apoptosis in NB cells, which is accompanied by reduced expression of the N‐Myc protein and LIN28B gene.^211^ This combination also reduces the expression of N‐Myc in tumor tissues and blocks tumor progression in vivo.^211^ Further, a study by Henssen et al.^254^ has shown that OTX015, a BRD inhibitor, reduces the viability in MYCN‐amplified NB cells, and shows potency against MYCN‐amplified NB xenografts. The same study has also shown that OTX015 has the potential to disrupt the BRD4‐chromatin interaction and suppress the expression of MYCN in NB cell lines.^254^

### 3.4 Necroptosis induction for NB therapy

Necroptosis is a form of cell death triggered by necrosis.^255^ It is driven by the interplay between receptors of necrotic death, their ligands, Toll‐like receptors (TLRs), interferons, and the necrosome complex.^22^ In normal cells, necroptosis is impeded via caspase‐8‐mediated cleavage of RIPK1/3 (receptor‐interacting serine/threonine‐protein kinase 1/3).^256^ Aggressive NBs frequently lack caspase expression, making them resistant to apoptosis. However, this can be exploited by inducing necroptotic cell death.^22^ Necroptosis can be induced in NB cells by increasing cytoplasmic Ca^2+^ to activate calcium‐calmodulin kinase II, which activates RIPK1.^257^ It is important to note that even NB tumors with decreased expression of caspase‐8 may be resistant to cell death instigated by drugs that induce necroptosis, possibly via epigenetic mechanisms.^258^ Demethylating agents or HDAC inhibitors may be used to induce cell death in chemoresistant NB tumors with defective caspase 8.

### 3.5 Targeting HIF in NB

In general, cancer cells are characterized by a hypoxic microenvironment, and such cells adapt to the hypoxic microenvironment via the upregulation of HIF, which mediates the transcription of several target genes that increase cancer progression.^259^ Various researchers have described a role for hypoxia in NB tumor initiation, cell survival, and metastasis.^60^ A study by Jögi et al.^15^ has shown that hypoxia stabilizes both HIF‐1α and HIF‐2α in NB cells. The same study has also demonstrated that pretreatment of SK‐N‐BE(2) NB cells in vitro at 1% O_2_ cause the tumor cells to have a reduced tumor latency and increases the growth of subcutaneous xenografts compared with SK‐N‐BE(2) cells pretreated under normoxic culture conditions.^15^ In addition, a study by Chen et al.^260^ has shown that HIF‐1α upregulation in NB promotes the proliferation, invasiveness, and migration of malignant cells via SHH signaling. HIF‐1α activation also provides resistance to antiangiogenic therapies in a xenograft mouse model of NB.^261^ Thus, inhibitors targeting HIF‐1α should be developed for NB therapy. Among the various inhibitors under development, PT2385 is a selective antagonist of HIF‐2 that has been found to decrease the expression HIF‐2 target genes and to inhibit tumor growth in a patient‐derived xenograft (PDX) model of clear cell renal cell carcinoma.^262^ Persson et al.^263^ have investigated the effects of inhibiting ARNT (aryl hydrocarbon receptor nuclear translocator)‐dependent HIF‐2 induced transcription by PT2385, and found that PT2385 treatment inhibits the dimerization between ARNT and HIF‐2α, and also reduces the nuclear HIF‐2α protein levels under hypoxia in the NB PDX. However, the same study has shown that there are no effects on HIF‐2 target gene expression, and no major change is observed in the cell survival in vitro or on the in vivo tumor growth after PT2385 treatment.^263^ In the same study, it has also been found that combination treatment using PT2385 with cisplatin or doxorubicin does not enhance the anticancer effects of the chemotherapeutic drugs compared with treatment with the chemotherapeutic drugs alone.^263^ Overall, more extensive studies should be conducted to identify new HIF inhibitors that might be useful as anticancer agents for NB treatment.

### 3.6 Targeting cancer exosomes in NB

Exosomes are small extracellular vesicles with a significant role in intercellular communication associated with cancer.^264^, ^265^, ^266^ There are many lines of evidence indicating that exosomes released from NB cells contribute to the progression of cancer. For instance, a study by Challagundla et al.^267^ has demonstrated a role for exosomes containing miRNAs in the resistance of NB to chemotherapy, wherein exosomal miRNAs such as miR‐21 and miR‐155 have critical roles in chemotherapy resistance through the TLR8‐NFкB and TERF1 signaling pathways. Another study by Haug et al.^268^ has shown that MYCN‐amplified NB cells release a variety of exosome‐like vesicular particles carrying various miRNAs (e.g., miR‐16, miR‐125b, miR‐21, miR‐23a, miR‐24, miR‐25, miR‐27b, miR‐218, miR‐320a, miR‐320b, and miR‐92a). The exosomal miRNAs released from MYCN‐amplified NB cells do not stimulate TLR8 signaling in recipient cells.^268^ However, a functional enrichment analysis reveals that NB exosomal miRNAs affect pathways relates to cell growth and cell death.^268^ These studies may indicate that targeting exosomes containing various miRNAs involved in the pathogenesis of NB may represent an effective approach to reduce NB tumorigenesis.

On the contrary, miR‐186 is responsible for the repression of oncogenic proteins (MYCN and AURKA) in NB cells, and is downregulated in NB and transforming growth factor‐β‐treated natural killer (NK) cells.^269^, ^270^ Thus, an approach to restore the miR‐186 levels in NB through NK cell‐derived exosomes could serve as another approach to reduce the tumor burden, promote survival, and restore the cell‐killing abilities of NK cells.^270^

## 4 CLINICAL STUDIES OF TARGETED NB THERAPY

During the last decade, several clinical trials of new monotherapies or combination protocols exist for high‐risk NB. The molecular targets of the drugs evaluated in clinical trials are depicted in Figures 2 and 3. The progress made in these trials, some of which are still ongoing, has streamlined the development of personalized medicine for children with high‐risk NB. Various combinations of small molecule inhibitors with standard chemotherapy or other agents have been tested for high‐risk NB patients, the details of which are summarized in Table 1.

**Table 1:** Overview of combination therapy being used in clinical trials for neuroblastoma

Inhibitor	Molecular target	Agent(s) used in combination	Phase	Status	Identifier	Results
Crizotinib	ALK	Topotecan hydrochloride	I	Completed	NCT01606878	Enrollment of 46 participants, and patients are assigned to experimental part A in which patients receive crizotinib, cyclophosphamide, and topotecan hydrochloride; part B in which patients receive crizotinib, vincristine, dexrazoxane, and doxorubicin, and part C in which patients receive crizotinib, cyclophosphamide, and topotecan hydrochloride) (posted on https://clinicaltrials.gov/) Eligible age for study—13 months to 21 years; and all sexes are eligible for the study (posted on https://clinicaltrials.gov/) Outcomes of this trial are divided into (a) primary outcome measures— the incidence of adverse events (summary of all toxicities), and maximum tolerated dose of crizotinib; and (b) secondary outcome measures —maximum plasma concentration, peak concentration (*C*_max_), terminal phase half‐life, area under the concentration, plasma clearance, and response rate (posted on https://clinicaltrials.gov/)
		Cyclophosphamide				
		Doxorubicin hydrochloride				
		Vincristine sulfate				
		Dexrazoxane hydrochloride				
Crizotinib	ALK	Standard therapy (busulfan, carboplatin, cisplatin, cyclophosphamide, dexrazoxane, doxorubicin, etoposide, isotretinoin, melphalan, thiotepa, topotecan, vincristine)	III	Recruiting	NCT03126916	Enrollment of 813 participants, and patients are assigned to the following experimental arms: (1) arm A (chemotherapy, hematopoietic stem cell transplantation (HSCT), external beam radiation therapy (EBRT); (2) arm B (chemotherapy, iobenguane I‐131, EBRT, HSCT); (3) arm C (chemotherapy, iobenguane I‐131, busulfan/melphalan [BuMel], EBRT, HSCT); arm D (chemotherapy, HSCT, EBRT); arm E (crizotinib, chemotherapy, HSCT, EBRT) (posted on https://clinicaltrials.gov/) Outcome for this trial is divided into—(a) primary outcome measures: event‐free survival (EFS) and (b) secondary outcome measures: incidence of adverse events, EFS, overall survival, response rate (posted on https://clinicaltrials.gov/) Eligible age for study—365 days to 30 years, and all sexes are eligible for the study (posted on https://clinicaltrials.gov/)
Ceritinib	ALK	Ribociclib	I	Recruiting	NCT02780128	EFS for patients with both F1174‐mutated ALK and amplified MYCN is significantly worse.^109^ Relapsed disease has a higher frequency of ALK mutations.^124^ Recurrent RAS/MAPK pathway mutations in tumors post‐ chemotherapy.^124^ Exposure of ribociclib is dose‐dependent at 350 (recommended phase II dose) and 470 mg/m^2^ (maximum tolerated dose) (equivalent to 600 (recommended phase II dose)—900 mg in adults).^271^
Lorlatinib	ALK	Chemotherapy (cyclophosphamide, topotecan)	I	Recruiting	NCT03107988	Enrollment of 40 participants, and includes the following experimental arms: (a) experimental cohort A1 (dose finding for lorlatinib); (b) experimental cohort A2 (adult and large BSA); (c) experimental cohort B1 (expansion); (d) experimental cohort B2 (combined with chemotherapy) (posted on https://clinicaltrials.gov/) Outcome measures for this trial: (a) primary outcome measures—RP2D (recommended phase 2 dose) of lorlatinib, toxicities of lorlatinib alone and in combination with cyclophosphamide and topotecan, pharmacokinetics (PK) (AUC for lorlatinib and metabolite, clearance for lorlatinib and metabolite, Cmax for lorlatinib and metabolite, Tmax for lorlatinib and metabolite, terminal half‐life for lorlatinib and metabolite) and (b) secondary outcome measures ‐evaluation of overall response, bone response, soft tissue response, and bone marrow response (posted on https://clinicaltrials.gov/) Eligible age for study—1 year to 90 years, and all sexes are eligible for the study (posted on https://clinicaltrials.gov/)
RG7388 (Idasanutlin)	MDM2	Chemotherapy (cyclophosphamide/topotecan/fludarabine/cytarabine/) or venetoclax	I/II	Recruiting	NCT04029688	Enrollment of 220 participants, and includes the following experimental arms: (a) dose‐escalation (idasanutlin single agent), (b) idasanutlin and venetoclax administration, (c) idasanutlin, cyclophosphamide/topotecan administration (posted on https://clinicaltrials.gov/) Outcome measures for this trial are—(a) primary outcome measures, which include the number of participants with at least one adverse effect and with dose‐limiting toxicities, percentage of participants with wild type *TP53* achieving objective response, and (b) secondary outcome measures include clinical benefit rate, duration of objective response, progression‐free survival, and percentage of participants with solid tumors achieving an objective response irrespective of TP53 status (posted on https://clinicaltrials.gov/) Eligible age for study—up to 30 years (child, adult), and all sexes are eligible for this study (posted on https://clinicaltrials.gov/)
Trametinib	MEK1/2	Dabrafenib (BRAF kinase inhibitor)	I/II	Recruiting	NCT02124772	Enrollment of 139 participants, and includes the following experimental arms—(a) trametinib dose‐escalation; (b) tumor‐specific expansion; (c) dose administration of trametinib in this part will be based on trametinib monotherapy RP2D from part a; (d) trametinib and dabrafenib dose administered in this part will be the combination of trametinib and dabrafenib RP2D from part C (posted on https://clinicaltrials.gov/) Primary outcome measures of this trial—safety assessment of trametinib via evaluating adverse events, ECG, ECHO (echocardiogram), changes in laboratory values, vital signs (posted on https://clinicaltrials.gov/) Secondary outcome measures of this trial—PK assessment of trametinib; and safety and tolerability assessment for trametinib for ECG, changes in laboratory values, and vital signs; tumor response for trametinib; effect of age and weight on PK of trametinib; PK and safety assessment of trametinib and dabrafenib when administered in combination (posted on https://clinicaltrials.gov/) Eligible age for study—1 month to 17 years (child), and all sexes are eligible for this study (posted on https://clinicaltrials.gov/)
Vorinostat	Histone deacetylase (HDAC)	Isotretinoin	I	Completed	NCT00217412	Enrollment of 60 participants, and includes the following experimental arms for NB patients: Arm I—patients receive vorinostat, and Arm III —patients receive isotretinoin and vorinostat (posted on https://clinicaltrials.gov/) Primary outcome measures include maximum tolerated dose and secondary outcome measures include the proportion of patients who demonstrate each polymorphism (posted on https://clinicaltrials.gov/) Eligible age for study—1 to 21 years, and all sexes are eligible for this study (posted on https://clinicaltrials.gov/)
Vorinostat	HDAC	Bortezomib	I	Completed	NCT01132911	Enrollment of five participants for this study, and the outcome measures include: (a) primary outcomes—determine the MTD (maximum tolerated dose) and (RP2D) recommended phase 2 dose of combination of bortezomib and vorinostat, and define toxicities and PKs; and (b) secondary outcomes—evaluate antitumor activity and determine biological activity of bortezomib via measurement of NFκB activity and endoplasmic reticulum stress response (posted on https://clinicaltrials.gov/) Eligible age for study—1 year to 21 years (child and adult) and all sexes are eligible for this study (posted on https://clinicaltrials.gov/)
Vorinostat	HDAC	^131^I‐MIBG	II	Active, but not recruiting	NCT02035137	Enrollment of 105 participants for this study, and includes arm A—^131^I‐MIBG alone; arm B—^131^I‐MIBG and irinotecan/vincristine; arm C—^131^I‐MIBG and vorinostat (posted on https://clinicaltrials.gov/) Primary outcome measures—overall response rate after treatment with the three arms mentioned above; and secondary outcome measures —compare toxicity profile associated with delayed engraftment, compare the occurrence of toxic death due to treatment regimens, and also compare toxicity profile for grade 3 or greater toxicities associated with treatment regimens (posted on https://clinicaltrials.gov/) Eligible age for study—2 years to 30 years, and all sexes are eligible for the study (posted on https://clinicaltrials.gov/)
Vorinostat	HDAC	Dinutuximab/GM‐CSF/IL‐2 and isotretinoin, and +/‐DFMO	II	Recruiting	NCT02559778	Enrollment of 500 participants for this study, and includes the experimental arm of standard immunotherapy with (arm B) and without (arm A) DFMO (posted on https://clinicaltrials.gov/) Primary outcome measures—measure the response of treatment based on EFS, and determine the feasibility of adding molecular targeted therapy to standard chemotherapy (posted on https://clinicaltrials.gov/) Secondary outcome measures —number of days that subjects remain alive, overall response rate after induction therapy, number of patients with treatment‐related adverse events, and amount of pain medicine required by arm A versus arm B (posted on https://clinicaltrials.gov/) Eligible age for the study—up to 22 years, and all sexes are eligible for the study (posted on https://clinicaltrials.gov/)
Vorinostat	HDAC	^131^I‐MIBG	I	Completed	NCT01019850	Enrollment of 27 participants for this study, and experimental arm includes the use of vorinostat, ^131^I‐MIBG, peripheral blood stem cell transfusion (posted on https://clinicaltrials.gov/) Primary outcome measure—identify all toxicities, and secondary outcome measure includes response evaluation in patients, and determine histone acetylation levels and norepinephrine transported mRNA levels in PBMCs after treatment with vorinostat (posted on https://clinicaltrials.gov/) Eligible age for study—2 years to 30 years (adult, child), and all sexes are eligible for the study (posted on https://clinicaltrials.gov/)
Vorinostat	HDAC	Isotretinoin	I	Completed	NCT01208454	Enrollment of 29 participants for this study, and includes experimental arm of isotretinoin and vorinostat treatment (posted on https://clinicaltrials.gov/) Primary outcome measure— all toxicities are determined and summarized in terms of type (organ affected or laboratory determination), severity; and also determine the maximum tolerated dose of vorinostat (posted on https://clinicaltrials.gov/) Secondary outcome measure —assess best response in solid tumors, survival and time‐to‐failure (posted on https://clinicaltrials.gov/) Eligible age for this study—up to 30 years (adult and child) and all sexes are eligible for the study (posted on https://clinicaltrials.gov/)
Decitabine	DNA methyltransferase (DNMT)	Doxorubicin and cyclophosphamide	I	Completed	NCT00075634	Enrollment of 21 participants for this study, and experimental arm includes treatment with decitabine, doxorubicin, cyclophosphamide, and G‐CSF (posted on https://clinicaltrials.gov/) Primary outcome measure—maximum tolerated dose (MTD) of decitabine and caspase 8 expression in bone marrow or tumor biopsy samples (posted on https://clinicaltrials.gov/) Secondary outcome measure —objective response rate, and percentage of apoptotic cells determined by TUNEL assay (posted on https://clinicaltrials.gov/) Eligible age for study—1 year to 21 years (adult and child), and all sexes are eligible for study (posted on https://clinicaltrials.gov/)
Gemcitabine(pyrimidine nucleoside analog)	DNA	Ribociclib	I	Recruiting	NCT03434262	Enrollment of 108 participants in this study, and this study includes the following experimental arms—(a) ribociclib and gemcitabine, (b) ribociclib and trametinib, and (c) ribociclib and sonidegib (posted on https://clinicaltrials.gov/) Primary outcome measure—estimate the RP2D/MTD of each arm, determine the PKs of combination treatment (posted on https://clinicaltrials.gov/) Secondary outcome measure —estimate response rate and duration of objective response of each arm (posted on https://clinicaltrials.gov/) Eligible age for this trial—1 year to 39 years (adult and child), and all sexes are eligible for the study (posted on https://clinicaltrials.gov/)
Gemcitabine(pyrimidine nucleoside analog)	DNA	Nab‐paclitaxel	I	Recruiting	NCT03507491	Enrollment of 24 participants in this study, and experimental arm includes treatment with gemcitabine and Nab‐paclitaxel (posted on https://clinicaltrials.gov/) Primary outcome measure—determine maximum dose tolerated of nab‐paclitaxel, and also toxicity of nab‐paclitaxel (posted on https://clinicaltrials.gov/) Secondary outcome measure —determine the anticancer activity of nab‐paclitaxel in combination with gemcitabine, evaluate the change in expression of SPARC (secreted protein acidic and rich in cysteine) in tumor, determine blood concentrations of paclitaxel (posted on https://clinicaltrials.gov/) Eligible age for study—6 months to 30 years (adult, child) and all sexes are eligible for the study (posted on https://clinicaltrials.gov/)
Etoposide	Topoisomerase	Monoclonal antibody 3F8	II	Completed	NCT00004110	Child, adult, older adult—all are eligible for this study, and all sexes eligible for this study (posted on https://clinicaltrials.gov/) Objectives—determine the antitumor effects of monoclonal antibody 3F8, etoposide, and isotretinoin; assess progression‐free survival (PFS) in patients; and also investigate the effects of oral etoposide on human antimouse Ab and anti‐idiotype response in patients (posted on https://clinicaltrials.gov/)
^131^I‐MIBG	Norepinephrine receptor	Carboplatin, etoposide, melphalan, and peripheral blood stem cell infusion, and radiotherapy	II	Completed	NCT00253435	28.57% is the poor‐risk patients with serious adverse events (posted on https://clinicaltrials.gov/) 37.5% is the good‐risk patients with serious adverse events (posted on https://clinicaltrials.gov/) Other (not including serious) adverse effects for poor‐risk patients include febrile neutropenia (73.81%), supraventricular and nodal arrhythmia (26.19%), diarrhea (76.19%), heartburn (7.14%), mucositis/stomatitis (oral cavity) (69.05%) (posted on https://clinicaltrials.gov/) Other (not including serious) adverse effects for good‐risk patients include febrile neutropenia (75%), ventricular arrhythmia (12.5%), mucositis/stomatitis (oral cavity) (62.5%), diarrhea (50%) (posted on https://clinicaltrials.gov/)
^131^I‐MIBG	Norepinephrine receptor	Dinutuximab	I	Recruiting	NCT03332667	Enrollment of 24 participants in this trial, and experimental arm includes treatment with ^131^I‐MIBG and dinutuximab (posted on https://clinicaltrials.gov) Primary outcome measures of this trial include—determination of MTD and RP2D of ^131^I‐MIBG and dinutuximab, and also define the toxicities of ^131^I‐MIBG in combination with dinutuximab (posted on https://clinicaltrials.gov) Secondary outcome measures include—evaluation of overall response, bone response, soft tissue response, and bone marrow response (posted on https://clinicaltrials.gov) Eligible age for study—1 year to 30 years (adult and child), and all sexes are eligible for the study (posted on https://clinicaltrials.gov)
^131^I‐MIBG	Norepinephrine receptor	Bevacizumab	I	Completed	NCT00450827	Enrollment of 25 participants for this clinical trial, and this study includes an experimental arm which involves the use of ^131^I‐3F8 and bevacizumab (posted on https://clinicaltrials.gov) Primary outcome measure— determine MTD (maximum tolerated dose) and age eligible for this study is 1 year and older (adult, child, older adult), and all sexes are eligible for this study (posted on https://clinicaltrials.gov)
Dinutuximab (ch14.18)	GD2	^131^I‐MIBG and nivolumab	I	Recruiting	NCT02914405	Enrollment of 36 participants for this clinical trial study, and the experimental arm includes a constant dose of ^131^I‐MIBG and the doses of ch14.18/CHO and nivolumab are determined by cohort I (nivolumab and no ch14.18/CHO), cohort II (50 mg/m^2^/cycle ch14.18/CHO and 3 mg/kg nivolumab), and cohort III (100 mg/m^2^/cycle ch14.18/CHO and 3 mg/kg nivolumab) (posted on https://clinicaltrials.gov) Primary outcome measures include—determine the safety and tolerability of ^131^I‐MIBG, ch14.18/CHO and nivolumab in pediatric patients; and secondary outcome measures include—antitumor response in patients receiving ^131^I‐MIBG, ch14.18/CHO and nivolumab in pediatric patients; and identify any association between KIR/KIR‐ligand genotype, FcγR genotype, and response (posted on https://clinicaltrials.gov) Eligible age for study – 1 year to 18 years (adult, child), and all sexes are eligible for this study (posted on https://clinicaltrials.gov)
Dinutuximab beta	GD2	Temozolomide and topotecan	II	Recruiting	NCT02308527	Enrollment of 224 participants in this trial, and the experimental arm includes temozolomide, temozolomide + bevacizumab, temozolomide + irinotecan, bevacizumab + temozolomide + irinotecan, topotecan + temozolomide, bevacizumab + topotecan + temozolomide, temozolomide + dinutuximab beta, dinutuximab beta + temozolomide + topotecan, dinutuximab beta + topotecan + cyclophosphamide (posted on https://clinicaltrials.gov) Primary outcome measures include—test the effect on bevacizumab addition to the chemotherapy (temozolomide, topotecan‐temozolomide, or irinotecan‐temozolomide) in NB patients, and evaluate the PFS; and secondary outcome measures —evaluate the toxicity of the different arms (posted on https://clinicaltrials.gov) Eligible age for study—1 year to 21 years (adult, child), and all sexes are eligible for the study (posted on https://clinicaltrials.gov)
Dinutuximab and sargramostim (GM‐SF)	GD2	Chemotherapy (carboplatin, cisplatin, cyclophosphamide, dexrazoxane, doxorubicin, etoposide, isotretinoin, melphalan, thiotepa, topotecan, vincristine)	II	Recruiting	NCT03786783	Enrollment of 45 participants in this study, and this study includes experimental arm (chemotherapy, dinutuximab, sargramostim, ASCT, EBRT) (posted on https://clinicaltrials.gov) Primary outcome measure includes toxicity rate and combined toxic death during treatment (posted on https://clinicaltrials.gov) Secondary outcome measures for this trial include—determine response rate, EFS, and overall survival (posted on https://clinicaltrials.gov) Eligible age for study—up to 30 years (adult and child) and all sexes are eligible for the study (posted on https://clinicaltrials.gov)
Dinutuximab	GD2	Immunotherapy (isotretinoin + sargramostim + IL‐2)	II	Active, not recruiting	NCT02169609	Enrollment of 25 participants in this trial study, and this study has primary outcome measures which include— assess the number of participants with serious and nonserious adverse effects, and on the contrary, secondary outcome measure includes—estimate the relapse‐free survival (posted on https://clinicaltrials.gov) Eligible age for study—adult, child, older adult, and all sexes are eligible for the study (posted on https://clinicaltrials.gov)
Isotretinoin	Unknown	Dinutuximab, aldesleukin, and sargramostim	III	Active, not recruiting	NCT00026312	Serious side effects of regimen A–RA only: cardiac disorders, eye disorders, nausea, vomiting, small intestinal obstruction, allergic reaction, anaphylaxis, lymphocyte count decreased (posted on https://clinicaltrials.gov). Serious side effects of regimen B (RA + immunotherapy): anemia, eye disorders, abdominal pain, diarrhea, fever, allergic reaction, anaphylaxis, sepsis, anorexia, hypokalemia, hyponatremia, urticaria, hypotension, respiratory, thoracic and mediastinal disorders, and vascular disorders (posted on https://clinicaltrials.gov). Immunotherapy (isotretinoin, GM‐CSF, ch14.18, and IL‐2) found superior to standard therapy (isotretinoin) in terms of rates of EFS.^32^
Hu14.18K32A	GD2	Cyclophosphamide and topotecan	II	Active, not recruiting	NCT01857934	Significant natural killer (NK) cytopenia caused due to chemoimmunotherapy, and complete recovery of NK cells takes place by the 21st day of each therapy cycle and autoHCT.^272^ Cytotoxicity of NK cells increased during treatment compared with diagnosis, and such cells conserve their ability to respond to cytokine stimulation.^272^ Two patients groups differ in therapy responses and primary tumor size were identified employing cluster analysis of CD56^bright^ NK cell count and tumor volume.^272^
Hu3F8	GD2	GM‐CSF	I/II	Recruiting	NCT01757626	Treatment with hu3F8 and GM‐CSF was outpatient without unexpected toxic effects and with reversible neuropathic pain.^273^ Maximum tolerated dose was not identified.^273^ Dose‐escalation was correlated to augmented serum levels and proceeded through a 9.6 mg/kg/cycle dosage.^273^ Out of 31 patients: (i) 5(16%) had stable disease, (ii) 14(45%) had partial response or complete remission, (iii) 1(3%) experienced dose‐limiting toxicity (DLT), and (iv) 11 (35%) showed early progressive disease.^273^
Hu3F8	GD2	Sargramostim	II	Active, not recruiting	NCT00072358	PRES (posterior reversible encephalopathy syndrome) was diagnosed in 2.3% of patients (5 of 215), including 2 of 55 patients who obtained HD‐3F8 and 3 of 160 patients who obtained SD‐3F8.^274^ PRES occurred in 3 of 26 patients (such patients’ prior treatment included external beam radiotherapy to the brain) compared with 2 of 189 patients (such patients did not receive prior brain irradiation).^274^ In 12 of 215 patients (5.6%), hypertension reached grade 3 toxicity, with 7 patients without PRES and 5 patients with PRES.^274^ Frequency of all five activation markers (CD11a, CD11b, CD63, CBRM1/5, CD87) was significantly higher in day 4 peripheral blood (PB) samples of cycle 1 of GM‐CSF plus anti‐GD2 Ab 3F8 as compared withday 0 PB samples.^275^ Progression‐free survival (PFS) is correlated to CBRM1/5‐positive granulocytes and increasing CBRM1/5‐positive granulocytes as positive prognostic factor for PFS.^275^
Hu3F8	GD2	Sargramostim	I	Completed	NCT00450307	Due to drug supply limitations, dose‐escalation stopped at 160 mg/m^2^/day, and there were no dose‐limiting toxicity (DLT), including absence of hypertension.^276^ Human antimouse antibody (HAMA) titer was uncommon if the treatment of 3F8 began less than 90 days after high dose alkylator‐based therapy.^276^ HAMA developed in approx. 40% of patients after one cycle of 3F8 if the initial 3F8 exposure occurred ≥90 days after high‐dose alkylator‐based therapy.^276^ Anti‐neuroblastoma activity was observed at all dosages, and mainly in those patients treated for refractory disease in comparison to progressive disease.^276^
Bevacizumab	VEGF‐A	Irinotecan and temozolomide	II	Completed	NCT01114555	Out of 34 participants, 3 patients (8.8%) have complete response, 18 patients (52.9%) have no response, 12 patients (35.3%) have progressive disease, and 1 patient (2.9%) was not treated (posted on https://clinicaltrials.gov/). Median overall survival (OS) and PFS were 31.5 ± 5.6 and 7.7 ± 1.7 months, respectively.^277^ Grade 4 toxicities include thrombocytopenia and neutropenia; and grade 3 toxicities were transaminitis, hepatic proteinuria, and diarrhea (3%).^277^
Bevacizumab	VEGF‐A	Cyclophosphamide and topotecan	II	Completed	NCT01492673	All‐cause mortality was 77.78% (7/9—affected/at risk) (posted on https://clinicaltrials.gov/) Serious adverse effects include febrile neutropenia, lymphocyte count decreased, platelet count decreased, and gastrointestinal disorders (posted on https://clinicaltrials.gov/)
C7R‐GD2. CART cells	GD2	Cyclophosphamide and fludarabine	I	Recruiting	NCT03635632	Enrollment of 94 participants in this clinical trial, and this trial has primary outcome measure which determines MRD (maximum tolerated dose) of C7R‐GD2.CART cells; and the secondary outcome measure is to determine the antitumor responses (posted on https://clinicaltrials.gov/) Eligible age for study—1 year to 74 years (adult, child, older adult), and all sexes are eligible for the study (posted on https://clinicaltrials.gov/)
Zoledronic acid	Farnesyl pyrophosphate synthase	Cyclophosphamide	I	Completed	NCT00206388	Enrollment of 21 participants in this study, and primary outcome measure includes to assess toxicity and determine maximum tolerated dose, and secondary outcome measures focused to investigate antitumor activity, and PKs in patients (posted on https://clinicaltrials.gov/) Eligible age for this study—up to 30 years (adult and child) and all sexes are eligible for the study (posted on https://clinicaltrials.gov/)
Zoledronic acid	Farnesyl pyrophosphate synthase	IL‐2	I	Terminated	NCT01404702	Enrollment of 4 participants in this study, and primary outcome measure is to evaluate the toxicity and safety of aldesleukin and zoledronic acid (posted on https://clinicaltrials.gov) Secondary outcome measures include—(1) evaluate the biologic function of autologous activated/expanded gamma delta T cells in NB patients receiving aldesleukin and zoledronic acid; (2) evaluate immune phenotype of in vivo activated/expanded autologous gamma delta T cells; and (3) evaluate tumor response in patients (posted on https://clinicaltrials.gov)
MLN8237	Aurora A kinase	Irinotecan and temozolomide	I/II	Completed	NCT01601535	Hematologic toxicities were common events, and patients were treated in phase II and oral solution cohorts.^278^ In phase II, four partial responses were observed in 19 patients, and the estimated PFS at 1 year was 34%. The PFS was 20% in the OS group.^278^ Alisertib oral solution had significantly higher median *C*_max_ at 45 mg/m^2^ compared with tablets at 60 mg/m^2^.^278^ Alisertib day 5 trough in the first cycle—associated with first cycle dose‐limiting toxicity (DLT).^278^ Patients having tumors with amplification of MYCN exhibited lower PFS as compared with patients without MYCN amplification.^278^
Difluoromethylornithine (DFMO)	Ornithine decarboxylase	Etoposide	I	Completed	NCT01059071	DFMO dose between 500 and 1500 mg/m^2^ orally twice a day exhibits no DLTs.^279^ A minor T‐allele at rs2302616 of the ODC gene in the patients had higher baseline urinary polyamine levels, and, the total urinary polyamines are decreased during first cycle of DFMO therapy.^279^ Patients having increased urinary polyamines exhibited less mean PFS.^279^ A minor T allele at rs2302616 of the ODC gene in the patients respond better to DFMO therapy as compared with individuals with major G allele at this locus.^279^
Difluoromethylornithine	Ornithine decarboxylase	Bortezomib	I/II	Active, not recruiting	NCT02139397	Enrollment of 16 participants in this study. This study has primary outcome measures which include—determine the tolerability and safety of DFMO in combination with bortezomib and also determine the overall response rate (ORR) (posted on https://clinicaltrials.gov) Secondary outcome measures include—determine the tolerability and safety of DFMO in combination with bortezomib; evaluate PFS; correlate PET scan with PFS; and correlate urinary polyamine levels with response and progression of NB disease (posted on https://clinicaltrials.gov)
Temsirolimus	mTOR	Temozolomide	II	Completed	NCT01767194	Of the 35 patients, 17 patients were assigned to irinotecan‐ dinutuximab‐temozolomide (arm 2), and 18 were assigned to irinotecan‐temsirolimus‐ temozolomide (arm 1).^280^ In arm 1, one patient achieved the partial response, and in arm 2, nine patients had objective responses, including five complete responses and four partial responses.^280^ Grade 3 adverse events in arm 1 were neutropenia, anemia, thrombocytopenia, increased alanine aminotransferase, and hypokalaemia.^280^ Grade 3 adverse events for arm 2 were pain, hypokalaemia, anemia, fever and infection, hypoxia, neutropenia, and thrombocytopenia.^280^ Arm 2 met the protocol criteria for selection as combination therapy for neuroblastoma whereas arm1 did not.^280^
Temsirolimus	mTOR	Perifosine	I	Completed	NCT01049841	Enrollment of 23 participants for this study; and the primary outcome measure includes—to determine the MTD of combination of temsirolimus and perifosine in patients (posted on https://clinicaltrials.gov) Secondary outcome measures include—(1) record the efficacy of perifosine and temsirolimus combination, and (2) determine whether PK serum levels of both temsirolimus and perifosine correlate with toxicity (posted on https://clinicaltrials.gov) Eligible age for study—up to 21 years (adult, child), and all sexes are eligible for the study (posted on https://clinicaltrials.gov)
Sorafenib	Multikinase inhibitor	Cyclophosphamide and topotecan	I	Active, not recruiting	NCT02298348	Enrollment of 18 participants in this study; and the primary outcome measures include— (1) determine the MTD (maximum tolerated dose) of sorafenib for pediatric patients, and (2) determine the number and type of toxicities of sorafenib when administered in combination with topotecan and cyclophosphamide (posted on https://clinicaltrials.gov) Eligible age for study—up to 30 years (adult and child) and all sexes eligible for this study (posted on https://clinicaltrials.gov)
Nifurtimox	DNA	Cyclophosphamide and topotecan	II	Active, not recruiting	NCT00601003	Enrollment of 112 participants in this study, and the primary outcome measure includes—assess the efficacy and safety of nifurtimox in combination with topotecan/cyclophosphamide in NB patients (posted on https://clinicaltrials.gov) Secondary outcome measures include—(1) assess the correlation between serum nifurtimox levels (in combination with topotecan/cyclophosphamide) with tumor response; (2) biology studies which focus on genome analysis of cells before and after treatment, biomarker development, and flow cytometry of tumor in bone marrow (posted on https://clinicaltrials.gov) Eligible age for study—up to 21 years (adult and child), and all sexes are eligible for this study (posted on https://clinicaltrials.gov)
Bortezomib	26S proteasome	Irinotecan	I	Completed	NCT00644696	Enrollment of 18 participants in this study, and the primary outcome measure is to determine the highest dose of IV irinotecan administered along with bortezomib without causing severe side effects (posted on https://clinicaltrials.gov) Secondary outcome measure includes—measure the NB tumors after treatment with bortezomib and irinotecan to determine any change in tumor size (posted on https://clinicaltrials.gov) Eligible age for this study include—1 year to 25 years (adult and child), and all sexes are eligible for the study (posted on https://clinicaltrials.gov)

### 4.1 Clinical trials of small molecule inhibitors

#### 4.1.1 ALK inhibitors

The ADVL0912 phase I/II trial involving crizotinib has been completed by the Children's Oncology Group (COG) in pediatric patients with solid tumors (NCT00939770).^281^ The early results of that trial have shown that out of 11 NB patients with known ALK mutations, one had a CR (complete response) and two patients had stable disease.^281^ Another phase I trial (NCT01606878) has been conducted in 2013 by the COG for anaplastic large‐cell lymphoma (ALCL) or high‐risk NB patients, to study the effects of crizotinib in combination with chemotherapy (dexrazoxane hydrochloride, topotecan hydrochloride, cyclophosphamide, doxorubicin, vincristine sulfate). A phase III trial (NCT03126916) is currently recruiting high‐risk patients to evaluate the effects of combining standard therapy with crizotinib. Another ALK inhibitor, ceritinib, has been examined in a phase I trial that assessed its efficacy as monotherapy against ALK‐activated pediatric malignancies, including NB (NCT01742286). In 2016, the clinical study Next Generation Personalized Neuroblastoma Therapy (NEPENTHE) was initiated and has been currently recruiting patients with NB (NCT02780128). The NEPENTHE study is placing patients in treatment groups on the basis of genetic aberrations identified using deep sequencing. Participants with ALK mutations are being treated with combination therapy using ribociclib and ceritinib. Another ALK inhibitor, ensartinib, has entered clinical trials of patients with non‐Hodgkin's lymphoma, relapsed or refractory NB, or histiocytic disorders with ROS1 or ALK genomic alterations (NCT03213652). Furthermore, a phase I trial of lorlatinib has been initiated by NANT (New Approaches to Neuroblastoma Therapy) Consortium, and in this trial, lorlatinib is either used as a single agent or combined with chemotherapy for relapsed or refractory NB patients (NCT03107988). NCI (National Cancer Institute) started a phase II trial that aims to classify patients into molecularly targeted treatments on the basis of genetic profiling (NCT03155620). In this study, ensartinib (ALK inhibitor) is being used for patients with relapsed or refractory NB (NCT03155620).

#### 4.1.2 MDM2 inhibitors

The p53‐MDM2 inhibitors have not been extensively explored at the clinical stage. The NIH U.S. National Library of Medicine website (https://clinicaltrials.gov/) provides data for RG7388 and HDM201, which have been used as MDM2 inhibitors for NB. The currently recruiting clinical trials for MDM2 inhibitors are focused on RG7388 (NCT04029688) and HDM201 (NCT02780128). The clinical trial of HDM201 (NCT02780128) is part of the NEPENTHE study described above. It is anticipated that some of the newer MDM2 inhibitors, such as RG7112,^219^ RITA,^282^ and SF1126,^283^ that are currently being investigated in preclinical studies for NB may enter clinical testing soon. Initial studies should focus on the pharmacokinetic properties in healthy individuals, and then test the potency in individuals with relapsed or refractory NB.

#### 4.1.3 RAS‐MAPK and MEK inhibitors

Several MEK inhibitors are currently being evaluated in pediatric patients. For instance, a phase I/IIa clinical trial (NCT02124772) is currently ongoing and recruiting patients to investigate the pharmacokinetic, safety, and clinical activity of trametinib monotherapy, and a combination of dabrafenib with trametinib, in cancer patients harboring V600 mutations. This study includes a patient population of *n* = 10 for individuals with refractory or relapsed NB to assess their response to mono‐ and combination therapy.

#### 4.1.4 PI3K/Akt/mTOR inhibitors

Different PI3K/Akt/mTOR inhibitors have been assessed in NB patients. For instance, the NANT Consortium has conducted a phase I clinical trial with SF1126 (inhibitor of PI3K and mTOR) for relapsed or refractory NB patients (NCT02337309). This trial had two pediatric phases. In the first phase, a dose‐escalation design of 3 + 3 was followed. In the second phase, once a recommended dose was identified, a population of 10 patients with MYCN amplified or Myc‐N expressing tumors was treated. In terms of AKT inhibitors, a clinical study has been conducted involving perifosine, an AKT inhibitor, for children with solid tumors (NCT00776867). This clinical study recruited 27 high‐risk NB patients, and only one patient had MYCN‐amplified high‐risk NB, while none of the tumors had an ALK mutation in 21 tested patients.^284^ A total of nine patients remained progression‐free for a median of 54 months from the study entry.^284^

#### 4.1.5 Targeting epigenetic regulators

BET (bromodomain and extraterminal domain) proteins are epigenetic readers, and bromodomains of BET proteins are capable to recognize histones and bind on them at acetylated lysine residues and thereby regulating the chromatin structure. Various BET inhibitors are in clinical trials. For instance, a phase I study of GSK525762 (I‐BET726) has been completed in March 2020. This study has evaluated the pharmacokinetics, safety, pharmacodynamics, and clinical activity of GSK525762 in patients with NMC (NUT Midline Carcinoma) and other cancers, including NB and MYCN‐driven solid tumors (NCT01587703). This study had age eligibility of 16 years and older, and all sexes were eligible for the clinical trial. At the epigenetic level, HDAC can be inhibited to increase acetylation and thus induce a less malignant transcriptional profile.^285^ Regarding HDAC inhibitors, a phase I clinical and pharmacokinetic trial study of vorinostat has been conducted in individuals with solid tumors including NB and was found to exhibit a maximum tolerated dose of 230 mg/m^2^/day.^286^ Vorinostat (class I and II HDACi) has been investigated in several trials in NB patients (NCT00217412, NCT01132911, NCT02035137, NCT02559778, NCT01019850, and NCT01208454). A varied phase II trial of combination therapy is also ongoing, that is, comparing treatment with ^131^I‐MIBG alone and vorinostat with ^131^I‐MIBG for resistant or relapsed NB (NCT02035137). Decitabine, a DNMT pan‐inhibitor, has been studied in a phase I trial in relapsed or refractory solid tumors or NB patients (NCT00075634). Another pan‐HDAC inhibitor, 4‐phenylbutyate (4PB), has also been evaluated in phase I clinical trial in patients with brain tumors or NB (NCT00001565).

#### 4.1.6 Clinical trials of nucleoside analogs and agents targeting DNA synthesis

One of the synthetic pyrimidine nucleoside analogs is gemcitabine that functions as a deoxycytidine triphosphate and incorporates into DNA strands synthesized during cell division. Various clinical trials are ongoing in the use of gemcitabine in NB patients. For instance, St. Jude Children's Research Hospital is recruiting patients for a phase I clinical trial to evaluate molecularly driven doublet therapies for individuals with various cancer conditions, including NB, and involves the use of drugs such as gemcitabine, ribociclib, sonidegib, and trametinib in doublets for patients (NCT03434262). Another phase I study is the one which is based on the combination treatment of gemcitabine with nab‐paclitaxel for relapsed and refractory pediatric solid tumors (NCT03507491). Another purine analog, fludarabine, has been evaluated in different clinical trials in NB patients.

#### 4.1.7 Clinical trials of selected immunotherapy regimens

Baylor College of Medicine, as a sponsor, has a completed phase I trial of a third‐generation GD‐2 chimeric antigen receptor (CAR) and iCaspase suicide safety study in NB patients (GRAIN) (NCT01822652). In this study, genes such as CD28 and OX40 are appended to GD2 T cells to make the cells live longer. One of the goals of the trial is to find out the highest safety dose of iC9‐GD2‐CD28‐OX40 (iC9‐GD2) T cells that can be provided to relapsed/refractory NB patients. The same study is also examining whether it is beneficial to give chemotherapy before the T‐cell infusion, which is referred to as lymphodepletion, and the chemotherapy used in this trial is a combination of cyclophosphamide and fludarabine. mAb therapy is also being combined with chemotherapy to treat NB, and a phase II trial has been conducted to study the effectiveness of the combination of a monoclonal anybody (3F8) against ganglioside GD2 and etoposide in patients with NB (NCT00004110).

#### 4.1.8 Clinical trials using ^131^I‐MIBG in NB

A norepinephrine analog is metaiodobenzylguanidine (MIBG), which is actively taken up via norepinephrine transporters and accumulates in NB cells. Radioiodine‐labeled MIBG can be used for the treatment and diagnosis of NB.^287^ This is because norepinephrine receptors are being targeted by MIBG, and such receptors are expressed in 90% of NB tumors.^288^ In addition,^131^I‐MIBG also holds the ability to target tumors irrespective of their MYCN status or specific histology.^289^ There have been several clinical trials of ^131^I‐MIBG for relapsed or refractory NB patients.^290^ The molecule has also been used in combination protocol with stem cell transplantation and chemotherapy, either as a single‐dose treatment or multiple‐dose treatment, for salvage treatment and as induction therapy for refractory or relapsed NB.^57^ U.S. FDA has approved ^131^I‐MIBG as a diagnostic agent in 1994 and for the imaging of pediatric NB in 2008.^291^ A dose‐escalation study has been performed by Matthay et al.^292^ in phase I trial of ^131^I‐MIBG to define its dose‐limiting toxicity with and without autologous bone marrow support in refractory NB patients. That study has found that a dose of up to 12 mCi/kg did not require autologous stem cell rescue, and 37% was the response rate at this particular dose.^292^ It has also found that doses up to 18 mCi/kg can be used safely, providing there is a possibility of autologous peripheral blood stem cell rescue.^292^ A large phase II trial has been performed to evaluate the effects of age, prior therapy, and disease site on the ^131^I‐MIBG therapy response to refractory NB patients.^293^ In that study, patients with (*n* = 148) and without (*n* = 16) cryopreserved HSCs (hematopoietic stem cells) have been treated with 18 and 12 mCi/kg of ^131^I‐MIBG, respectively. The ORR (overall response rate) was 36%, and also the response rate in patients older than 12 years was significantly higher.^293^

Apart from monotherapy, various combinations have been studied, such as the use of ^131^I‐MIBG with chemotherapeutic agents like cisplatin,^294^, ^295^ topotecan,^296^ cyclophosphamide,^297^ and melphalan,^298^ which yielded response rates of 27%–80%.^294^, ^295^, ^299^ Phase II study has investigated the effects of ^131^I‐MIBG in combination with chemotherapy (carboplatin, etoposide, melphalan, referred to as CEM) and radiation therapy in patients acquiring bone marrow transplants or autologous peripheral stem cell for relapsed or refractory NB (NCT00253435).^289^ In that study, a 10% response rate was observed in primary refractory or progressive disease patients, and the addition of ^131^I‐MIBG to CEM was found to be tolerable for high‐risk NB patients. Another study by Mastrangelo et al.^297^ has shown that patients who received chemotherapy accompanied by treatment with ^131^I‐MIBG (200 mCi) for relapsed and refractory NB has impressive response rates, where five patients having treatment‐naive had one complete response, two very good partial responses, and two partial responses, thus suggesting that ^131^I‐MIBG has particular promise for the treatment of previously untreated individuals. ^131^I‐MIBG is currently being evaluated in the upfront treatment of patients with HR‐NB in a COG‐NCI‐sponsored Phase III clinical trial (NCT03126916).^300^

#### 4.1.9 Immunotherapy‐based clinical trials

##### 4.1.9.1 Monoclonal antibodies

Dinutuximab is a chimeric mAb and composed of human constant regions of IgG1 and murine variable regions of IgG3, targets glycolipid GD2 in NB cells. Dinutuximab was FDA approved in 2015 to treat high‐risk NB patients.^9^ Various clinical trials are active involving dinutuximab for patients with NB (NCT02743429, NCT04253015, NCT03332667, NCT02169609, NCT02914405, NCT04221035, NCT02308527, NCT00026312, NCT01041638, NCT03786783, NCT02914405, NCT01711554, NCT02169609, NCT02169609, and NCT04253015). Dinutuximab therapy has various side effects, such as fever, neuropathic pain, hypotension, and allergic reactions.^301^ In terms of their mechanism of action, anti‐GD2 mAbs employ both antibody‐dependent cell‐mediated cytotoxicity (ADCC) and complement‐dependent cytotoxicity to exert its effects in cancer patients.^302^ Hu14.18K322A is a humanized form of dinutuximab, and currently in a phase II trial sponsored by St. Jude Children's Research Hospital for advanced stage NB. It is being given with IC, and early results suggest that it can significantly improve the early response, reduce the volume of tumor, and improve the 2‐year EFS (NCT01857934). Another mAb, enoblituzumab, targets B7‐H3 (a type I transmembrane glycoprotein). It has been used in a phase I, open‐label trial to characterize antitumor activity of enoblituzumab in young adults and children expressing B7‐H3 in relapsed or refractory malignant solid tumors (including NB) (NCT02982941). Naxitamab (hu3F8), a humanized mAb targeting ganglioside GD2, has also been tested in NB patients (NCT01419834, NCT01757626, and NCT03033303). These trials of hu3F8 have been demonstrated that it has favorable pharmacokinetics, low immunogenicity, and improved toxicity profile.^273^, ^303^, ^304^ Bevacizumab (Avastin) is a humanized mAb that targets VEGF, and inhibits tumor growth.^305^ This mAb has been tested in phase I and II trials for relapsed or refractory NB (NCT01114555, NCT00885326, NCT00450827, NCT02308527, and NCT01492673). Among the following trials, NCT00885326, NCT00450827, and NCT02308527 have no published results. However, a phase II trial of the combination of bevacizumab, irinotecan, and temozolomide (BIT) in high‐risk NB patients (NCT01114555) has shown 3 patients with complete response (CR), 12 with progressive disease, and 18 with no response. The OS and median progression‐free were 31.5 ± 5.6 and 7.7 ± 1.7 months, respectively. Grade 4 toxicities in patients were thrombocytopenia (24%) and neutropenia (30%), grade 3 toxicities include proteinuria (9%), diarrhea (3%), and hepatic transaminitis (15%).^277^ Another trial using the combination of cyclophosphamide, topotecan, and bevacizumab in NB patients (NCT01492673) showed the all‐cause mortality was 66.67%, and serious adverse events include febrile neutropenia (66.67%), and other adverse effects include anemia (11.11%), diarrhea (22.22%), metabolism and nutrition disorders, cystitis noninfective, hematuria and alopecia.

##### 4.1.9.2 Conjugated antibodies

Antibodies can be conjugated with therapeutic agents (radionucleotides, drugs, and cytokines) to enhance their functions against cancer cells. An example of the antibody‐drug conjugate is lorvotuzumab mertansine (IMGN901), which is composed of a humanized anti‐CD56 antibody linked to tubulin‐binding maytansinoid DM1, and is evaluated in an active (but not recruiting) phase II trial for patients with relapsed or refractory solid tumors, including NB (NCT02452554). Hu14.18‐IL2 (EMD273063) is a genetic fusion of interleukin‐2 (IL‐2) attached to the carboxy terminus of each IgG heavy chain of Hu14.18. Hu14.18‐IL2 has been part of various clinical trials, some still ongoing, in NB patients (NCT03209869, NCT00082758, NCT01334515, and NCT00003750).

#### 4.1.10 Targeting the tumor microenvironment (TME) in NB

For therapeutic intervention, clinical trials are following three strategies to target the TME, and include the following:

##### 4.1.10.1 Targeting TME cells

Zoledronic acid (ZA) targets osteoclasts in the TME by inhibiting farnesyl pyrophosphate synthase. New Advances in Neuroblastoma Therapy consortium has conducted phase I trial involving ZA and demonstrated that zoledronic is safe to use in individuals with bone metastasis. In addition, ZA has been evaluated in combination with cyclophosphamide in a phase I study (NCT00206388), and in combination with IL‐2 in another clinical trial (NCT01404702). Endothelial cells are also key targets in clinical trials, and a phase I trial has been conducted using bevacizumab (molecular target is VEGF), ZA, and cyclophosphamide in patients with recurrent or refractory high‐risk NB (NCT00885326).

##### 4.1.10.2 Targeting signaling pathways activated by the TME

Small molecule inhibitors are being used to target signaling pathways that are activated by TME. For instance, sorafenib, a multikinase inhibitor, is used in combination with cyclophosphamide and topotecan in NB patients (NCT02298348). Lestaurtinib (CEP‐701) (inhibitor of TrkA/B/C and Janus kinase 2 (JAK2)) has been tested in individuals with recurrent or refractory high‐risk NB (NCT00084422). Further, ZD6474 (a VEGFR inhibitor) has been tested alone and in combination with the retinoic acid in a phase I trial in pediatric NB patients (NCT00533169). An inhibitor of MEK/MAPK signaling kinase and topoisomerase II‐β, (R+) XK469), has been evaluated in a phase I trial in patients with advanced NB (NCT00028522). Temsirolimus is an inhibitor of Akt pathway that targets mTOR protein and undergoes a phase II trial in individuals with relapsed or refractory NB (NCT01767194).

##### 4.1.10.3 Chimeric antigen receptor (CAR)  T cells

Apart from monoclonal Abs, CAR T cells are developed to target GD2, and the efficacy of such cells has been evaluated in clinical trials. Several clinical trials (NCT02919046, NCT03373097, NCT03635632, NCT02761915, NCT02765243, NCT02311621, NCT02107963, and NCT01822652) have examined the application of CAR T cells in NB patients. Ganglioside GD‐2 is being targeted as a part of immunotherapy‐based approaches for NB. GD‐2 has been targeted by various monoclonal antibodies, such as 3F8, in the combination protocol with sargramostim (NCT00072358), or granulocyte‐macrophage colony‐stimulating factor (NCT00450307). In addition, activated T cells are being armed with GD2‐bispecific antibody, and are being evaluated in phase I/II trials in young adults and children with NB (NCT02173093).

#### 4.1.11 Radionucleotide therapy

DOTATATE is an amino acid peptide with a covalently‐bonded DOTA bifunctional chelator that can also be bound to gallium‐68 and lutetium‐177 to form ^68^Ga‐DOTATATE and ^177^Lu‐DOTATATE. This radiolabelled DOTATATE has been used in NB, which expresses somatostatin receptor‐positive, but does not express NET (norepinephrine transporter) or exhibits MIBG resistance.^56^ In January 2018, ^177^Lu‐DOTATATE was approved by FDA for neuroendocrine tumors, and currently in clinical trials for therapy for NB tumors.^56^, ^306^ A phase III trial of ^177^Lu‐DOTATATE (NCT01578239) has been demonstrated marked improvements in the EFS of neuroendocrine tumor‐bearing patients in comparison to those treated with long‐acting repeatable octreotide.^307^ ^177^Lu‐DOTATATE has also been found to be effective on its use with radiosensitizing agents such as topotecan and nutlin‐3.^308^ In addition, an ongoing phase II trial is being performed to assess the efficacy of ^68^Ga‐DOTATATE as a diagnostic test in patients with somatostatin receptor‐positive NB tumors (NCT03273712).

#### 4.1.12 Other agents

Studies have shown that inhibition of Aurora can destabilize the MYCN. MLN8237 is an Aurora A kinase inhibitor, and was used in combination with temozolomide and irinotecan in a phase I/II trial for NB (NCT01601535). Another Aurora kinase inhibitor, alisertib, has been used as monotherapy in phase I and II trials for NB patients (NCT02444884, NCT01154816).

DFMO (eflornithine) is an inhibitor of ornithine decarboxylase, and has been examined in a phase I trial in both alone conditions and also in combination with etoposide (NCT01059071).^279^ The same trial has also studied the dose‐limiting toxicity of DFMO, and found that 500–1500 mg/m^2^/day doses of DFMO are safe and well‐tolerated in relapsed NB patients.^279^ Further, NCT02139397 is a currently active but not recruiting phase I/II trial and this trial will evaluate the combination of bortezomib with DFMO for relapsed or refractory NB.

Entrectinib, a Trk inhibitor, has been evaluated in a phase I trial (STARTRK‐1) to assess its tolerability and safety, and to determine the recommended dose in patients with various malignancies, including NB (NCT02097810). In addition, phase I and II trials of entrectinib are currently ongoing for individuals with solid tumors, including neuroendocrine tumors and NB (NCT02650401 and NCT02568267). The effectiveness of mTOR inhibitors alone and in combination with other treatments have also been investigated. For instance, temsirolimus (mTOR inhibitor) has been tested in combination with temozolomide in clinical trials for NB patients (NCT01767194). Combining an inhibitor of AKT signaling with mTOR inhibition has been found to be more effective than either treatment alone; for instance, a phase I clinical trial has studied the safety and effectiveness of perifosine (AKT inhibitor), and temsirolimus (mTOR inhibitor) in recurrent pediatric solid tumors (NCT01049841).

Sorafenib is a multikinase inhibitor with activity against many protein kinases, such as VEGFR, PDGFR, and RAF. A phase I trial of sorafenib together with topotecan and cyclophosphamide is currently active for relapsed and refractory NB patients (NCT02298348). An antiparasitic agent, nifurtimox, has been used in the treatment of Chagas disease. This drug is tested in a phase II trial, and in this trial, nifurtimox has been used in combination with topotecan and cyclophosphamide for the treatment of relapsed or refractory NB (NCT00601003). The molecular target of Nifurtimox has not been fully elucidated in NB. A study by Sholler et al.^309^ has shown that nifurtimox treatment induces the formation of reactive oxygen species, causes DNA fragmentation, and suppresses Akt phosphorylation in NB cells, leading to apoptosis. Another study by Stanchi et al.^310^ has shown that nifurtimox can reduce N‐myc expression in NB cells. A study by Kong et al.^311^ has indicated that nifurtimox causes deactivation of Akt‐GSK‐3β signaling in NB cells. Different research groups have proposed different mechanisms of anticancer action for nifurtimox; the exact mechanism(s) underlying the cytotoxicity of nifurtimox warrants further investigations. Gefitinib, an EGFR inhibitor, underwent a phase I trial in patients having refractory solid tumors, including NB (NCT00132158). A proteasome inhibitor, bortezomib, has also been investigated in individuals with recurrent or refractory NB (NCT00644696).

As described above, several new drugs/strategies are currently being investigated in patients with NB. However, the antitumor activity observed for these targeted therapies has not been as promising as expected, so it is important to focus on obtaining a comprehensive understanding of the molecular mechanisms involved in the relapse of NB and the lack of the response to existing therapies. Further, most of the existing therapies have been tested in unselected populations, so patient selection methods should be improved to develop more specific, accurate, and efficient therapies for NB.

## 5 GENERAL DISCUSSION AND FUTURE RESEARCH DIRECTIONS

NB is a heterogeneous disease with varied outcomes, ranging from spontaneous regression to refractory growth. Thus, the most pressing need is to develop novel and effective strategies for the treatment of NB. To improve the clinical outcome for children, it is critical to understand the etiology of NB. Genome analyses suggest that chromosome instability (CIN) is a significant event in the pathogenesis of NB.^4^ However, further investigations are needed to confirm whether CIN is the root cause of the gene mutations observed in NB. It is also crucial to understand the cause of CIN in NB and investigate the importance of aberrations in proteins involved in chromosome and centrosome segregation, spindle apparatus machinery, and DNA repair.^4^ One of the recurrent genetic alteration observed in the high‐risk NB is the hemizygous deletion of the chromosome 11q 22‐23.^312^ ATM is located on the arm of chromosome 11, and has a role in DNA repair,^312^, ^313^, ^314^ and its loss may be related to the induction or progression of NB. It may be useful to inhibit PARP (poly ADP ribose polymerase) to target malignancies that exhibit homologous recombination DNA repair pathway deficiencies.

The currently available targeted therapy for NB includes: (1) targeting genetic aberrations; (2) targeting disrupted signaling molecules; (3) immunology‐based approaches; (4) radiopharmaceutical targeting of norepinephrine and somatostatin receptors; (5) targeting epigenetic regulators; and (6) targeting Bcl‐2 family proteins. Among these strategies, cancer immunotherapy has emerged as an effective strategy. It is also associated with less acute or chronic toxicity than genotoxic therapies. The humanization and modification (by point mutations) of the GD2 antibody are expected to reduce the immunogenicity and side effects of the treatment.^9^ For instance, the *O*‐acetylated form of GD2 reduces off‐tumor target effects on mesenchymal stromal cells, sensory neurons, and the posterior lobe of the pituitary gland.^9^ New antibody formats should be developed to deliver high‐dose radiation to cancer cells, reducing toxicities.^61^ CAR T cells have also been shown to induce clinical response in NB patients. However, they have only limited success due to difficulties with target antigen selection, a lack of T‐cell persistence, and a suppressive TME.^58^ Thus, CAR T cells should be engineered to address the barriers present in cancer patients. Single‐cell RNA sequencing of NBs should also be implemented to identify new leads for immunotherapeutic strategies.^315^ Very few compounds targeting epigenetic factors (i.e., HDACi, DNMTi, iBET) have entered clinical trials. As a future direction, the crystal structures of epigenetic regulators should be resolved to develop better inhibitors with fewer side effects. In addition, combination therapy should also be developed by targeting different sites of a pathway, or with the intention of disrupting multiple pathways together.

As a future direction, new therapeutic approaches should be developed to reduce the risk of NB disease progression. Among the new approaches being developed is HIFU (high‐intensity focused ultrasound).^316^ Similarly, MR (magnetic resonance)‐HIFU has been developed for the treatment of NB, in which HIFU functions to ablate tumor lesions under the real‐time anatomic guidance and thermal monitoring of MRI (magnetic resonance imaging).^317^ MR‐HIFU has been implemented in trials for individuals with relapsed/refractory solid tumors. For instance, AeRang Kim (sponsored by the Children's National Research Institute, Washington, DC) is currently recruiting patients for a phase I study to evaluate the feasibility and safety of MR‐HIFU therapy for individuals with relapsed or refractory solid tumors, including NB (NCT02076906). Further, the same group is recruiting patients for a phase I study to determine the MTD (maximum tolerated dose) and RP2D (recommended phase 2 dose) of LTLD (lyso‐thermosensitive liposomal doxorubicin) administered in combination with MR‐HIFU for patients with relapsed/refractory solid tumors, including NB (NCT02536183). Research has also been conducted to evaluate the anatomic locations of NB tumors to determine the feasibility of MR‐HIFU therapy.^318^ In one study, it has been found that only a minority of NB tumors are treatable at the time of diagnosis or relapse, and at diagnosis, all treatable NBs are intra‐abdominal and not‐targetable without the use of respiratory motion compensation.^318^ It has also been found that at relapse, many NB patients have intrathoracic, intracranial, or intraabdominal tumors that are not targetable due to adjacent anatomical structures.^318^ Thus, the use of MR‐HIFU for drug delivery in NB is primarily limited to a fraction of relapsed tumors, as only a minority of tumors are targetable at diagnosis.^318^ To improve the technology of MR‐HIFU, 3D modeling using the MR‐HIFU system should be implemented to improve its accuracy in predicting targetable lesions.^318^

At the preclinical level, a study by Eranki et al.^319^ has shown that a combination of mechanical HIFU fractionation and checkpoint inhibitors (αCTLA‐4 + αPD‐L1) significantly enhances the systemic antitumor response compared with a previously unresponsive murine NB model, providing evidence that HIFU may have synergistic potential with immunotherapy for the treatment of NB. In addition, combining MR‐HIFU with existing chemotherapy should be implemented to augment the chemoresponse of NB, and increase local control and decrease systemic toxicity.^318^

Hyperthermic temperatures also have exhibited therapeutic potential in various applications, including drug/gene delivery and radio/chemosensitization, and such temperatures can be generated in biological tissues by applying pulsed focused ultrasound.^320^ Experiments based on theoretical simulations, in vitro validations, and an in vivo subcutaneous murine xenograft model have been conducted in the past to optimize and select pulsed‐focused ultrasound (FUS) exposure parameters for hyperthermia‐based applications. Such experiments can help reduce animal experimentation and are easy to into clinical trials since they are noninvasive or minimally‐invasive.^320^ In addition, hyperthermia has also been found to be effective in enhancing the anticancer activity of chemotherapeutic drugs.^321^ This is supported by a study carried out by Debes et al.^322^ which showed that hyperthermia synergistically enhances the cytotoxicity of an alkylating agent, cisplatin, in NB cells. Targeted image‐guided drug delivery (IGDD) is a technique that has been developed to improve drug deposition in a variety of diseases, including cancer.^323^ IGDD employs focused ultrasound along with “microbubble” ultrasound contrast agents (UCAs) to enhance drug delivery into target tissues.^323^ This approach is known as sonopermeation, which helps to increase vascular permeability and thus increased penetration of drugs into the target tissue.^323^ A report by Bellary et al.^324^ has described a novel 2D and 3D quantitative contrast‐enhanced ultrasound imaging (qCEUS) system that could be used to monitor the therapeutic efficacy of sonopermeation in NB tumors. That study has also found that combing ultrasound therapy with UCA significantly enhances the doxorubicin payload to NB in an orthotopic xenograft model, and qCEUS imaging indicates that there is significant doxorubicin uptake induced by increasing the tumor vascular permeability (reduced pericyte coverage) due to microbubble sonopermeation, with no damage to vasculature.^324^ Overall, ultrasound‐based approaches already have a significant impact on the diagnosis and management of NB, and may also be useful for the treatment of NB.

Currently, a significant gap exists between in vitro and in vivo testing to identify new drugs for NB. As is typical for drug development, only about 1 in 10 drugs tested in clinical trials are finally approved by the FDA.^45^ The models being used for NB research and drug efficacy studies include zebrafish, mice, and chick chorioallantoic membrane (CAM).^325^ Using a more physiologically‐relevant and technically‐reproducible model system would facilitate the development of treatments for NB and other disease states.^325^ One such system is the 3D tissue‐engineered system, which can model the TME to provide a better understanding of the NB pathogenesis, and also test a drug in more relevant conditions. Further, a patient‐derived xenograft model system can be used to screen and identify effective drugs for NB treatment. The PDX model has high human relevance in terms of exhibiting features of human NB such as the presence of human stromal cells to mimic the TME, genetic complexity, histopathology, and mutational and proteomic profiles of actual patient tumors.^325^ With regard to using PDX models in cancer research, a pediatric preclinical testing program (PPTP) has been entrenched to identify effective treatments for pediatric cancers. The aim of this program is to acquire preclinical in vivo data with the help of genomically‐characterized PDX cell lines to test and identify agents that can easily advance in pediatric clinical trials (NCI PPTC, www.ncipptc.org).^326^ As a future direction, patient‐derived NB cells should be incorporated in 3D culture systems to test and identify new effective drugs and combinations that can then be tested in PDX models of NB, or in the case of drugs already approved for other indications, in human clinical trials.

Regarding the treatment of high‐risk NB, the patient selection for clinical trials of molecular targeted therapy should be improved by considering tumor heterogeneity.^25^ In addition, novel statistical methods should be considered in early phase trials to minimize the requested number of patients, making it easier to recruit a sufficient number of NB patients to analyze.^25^ Issues associated with the tumor heterogeneity in NB may be overcome by studying liquid biopsies for cell‐free DNA.^25^ This approach would help to detect genetic aberrations that may not be identified by studying a single site of disease, and would ameliorate the need for a tissue biopsy.^25^

Overall, it is important to understand the genetic complexity of NB and to develop personalized medicine based on the genetic predisposition of individual patients. Continued attempts should be made to identify new signaling pathways critical for the pathogenesis of NB and resistance to treatment, which can be further exploited as targets for therapy. There is also a need to identify prognostic tumor markers that could be used to assess the prognosis of patients and their response to treatment. The ultimate goal is to apply all of this knowledge to cure children with NB with minimal toxicity.

## CONFLICT OF INTERESTS

The authors declare that there are no conflict of interests.

## AUTHOR CONTRIBUTIONS

*Study concept and design*: Wei Wang, Jia Zhou, Frank McKeon, Jennifer Foster, and Ruiwen Zhang. *Drafting of the manuscript*: Atif Zafar, Wei Wang, Xinjie Wang, Gang Liu, Jennifer Foster, and Ruiwen Zhang. *Revising of the manuscript*: Wei Wang, Gang Liu, Xinjie Wang, Frank McKeon, Jennifer Foster, Jia Zhou, and Ruiwen Zhang. *Administrative, technical, or material support*: Wei Wang and Ruiwen Zhang. *Study supervision*: Wei Wang and Ruiwen Zhang. All the authors have read and agreed to the published version of the manuscript.
